# Thresholds of Oxidative Stress in Newly Diagnosed Diabetic Patients on Intensive Glucose-Control Therapy

**DOI:** 10.1371/journal.pone.0100897

**Published:** 2014-06-27

**Authors:** Rashmi Kulkarni, Jhankar Acharya, Saroj Ghaskadbi, Pranay Goel

**Affiliations:** 1 Department of Biology, Indian Institute of Science Education and Research, Pune, Maharashtra, India; 2 Department of Zoology, University of Pune, Pune, Maharashtra, India; 3 Mathematics and Biology, Indian Institute of Science Education and Research, Pune, Maharashtra, India; St. Vincent's Institute, Australia

## Abstract

Cellular and animal studies suggest that oxidative stress could be the central defect underlying both beta-cell dysfunction and insulin resistance in type 2 diabetes mellitus. A reduction of glycemic stress in diabetic patients on therapy alleviates systemic oxidative stress and improves insulin resistance and beta-cell secretion. Monitoring oxidative stress systematically with glucose can potentially identify an individual's recovery trajectory. To determine a quantitative model of serial changes in oxidative stress, as measured via the antioxidant glutathione, we followed patients newly diagnosed with diabetes over 8 weeks of starting anti-diabetic treatment. We developed a mathematical model which shows recovery is marked with a quantal response. For each individual the model predicts three theoretical quantities: an estimate of maximal glutathione at low stress, a glucose threshold for half-maximal glutathione, and a rate at which recovery progresses. Individual patients are seen to vary considerably in their response to glucose control. Thus, model estimates can potentially be used to determine whether an individual patient's response is better or worse than average in terms of each of these indices; they can therefore be useful in reassessing treatment strategy. We hypothesize that this method can aid the personalization of effective targets of glucose control in anti-diabetic therapy.

## Introduction

Fasting plasma glucose (FPG) and glycated hemoglobin (HbA_1C_) are the central measures of diagnosis of Type 2 diabetes (FPG ≥126 mg/dl (7.0 mmol/L); HbA_1C_ ≥6.5%) and prediabetic states (FPG 100–125 mg/dl (5.5–6.9 mmol/L); HbA_1C_ 5.7–6.4%). The clinical management of diabetes focuses on the control of hyperglycemia using a combination of nutritional and pharmacological therapies. Beginning with the current (2013) recommended Standards of Medical Care in Diabetes the ADA recommends a “patient-centred and personalized care” regimen to determine appropriate targets of glycemic control. The benefits of tight glucose control have to be weighed in relation to its risks; in the context of variety of factors that include prevailing health risks such as long duration of disease or comorbidities, personal preferences and other social and economic considerations it may be appropriate to relax HbA_1C_ targets^1^ to 7.5–8.5% (58.5–69.4 mmol/mol). There is thus great interest in asking how phenotypic, genotypic or pathophysiological characteristics of a patient might guide the personalization of their therapy [1], [2].

Beta-cell dysfunction and insulin resistance (IR) together underlie the development of diabetes, although there may be differences between their relative contributions in Asian and Westernized populations [2], [3]. The development of insulin resistance is the primary event in the metabolic syndrome; with time, if beta-cell failure occurs as well, these results in frank hyperglycemia. The etiology of the development of insulin resistance is complex and not fully understood. However, compelling cellular and *ex vivo* tissue models have indicated a causal role for oxidative stress (OS) in the development of IR [4]–[11]. In humans, an association between OS that arises from chronic overnutrition and physical inactivity and IR has been observed in individuals with impaired fasting glucose [12], [13], but a relationship between them has not been unambiguously established. Apart from its action on insulin sensitivity, hyperglycemia also exerts a direct negative effect on beta-cell function (“glucotoxicity”). Hyperglycemia-induced OS has been clearly indicated in deterioration of beta-cell function [14]; controlling OS either by using antioxidants [15], [16] or by overexpressing antioxidant enzymes [17], [18] restores beta-cell function.

Biophysically, hyperglycemia generates reactive oxygen species (ROS) in excess [11]: it can therefore be expected that controlling hyperglycemia would improve an OS state of the cell as well. Thus, if oxidative stress is causally implicated in the development of beta-cell dysfunction and insulin resistance, then it ought to be useful to monitor oxidative stress in diabetic patients. In particular, if pathophysiological differences between individuals in the oxidative stress response to glycemic stress can be expressed quantitatively, that can potentially be useful (a) in determining the extent of progress of the therapy, and (b) adjusting an appropriate glycemic target for a patient.

**Figure 1:**
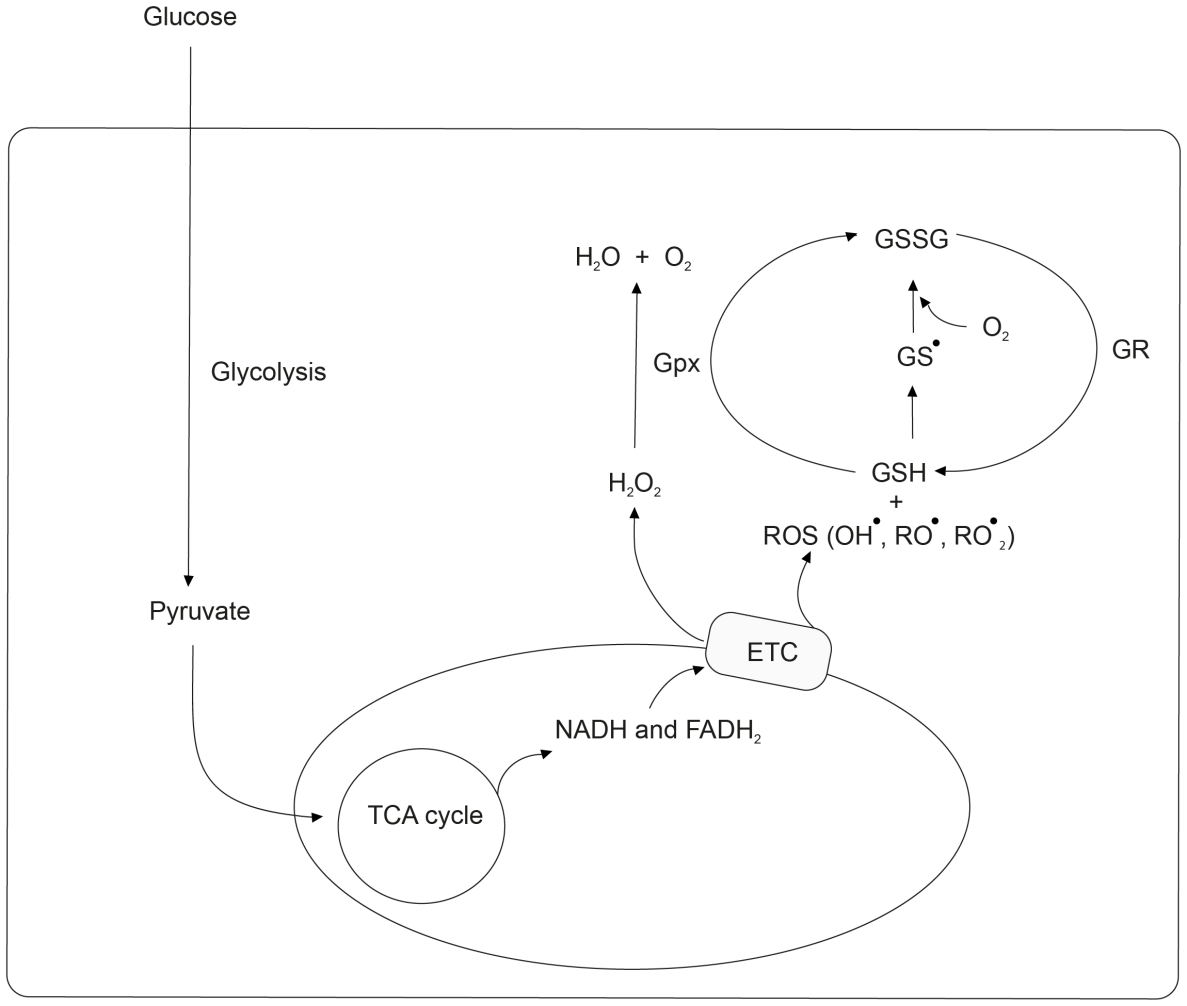
Glutathione redox reactions. A major role of GSH in the cellular antioxidant defense network is to neutralize ROS produced during glycolysis; a partial subset of these reactions is shown in relation to the GSH-GSSG couple.

We have previously shown by measuring multiple biomarkers of OS serially across 8 weeks that OS improves concomitantly with lowering glucose in diabetic patients on treatment [19]. Over twelve different biomarkers were studied, each of which improved with glucose control; of these glutathione appears to respond rapidly and in strong association with changes in glucose. Glutathione is an endogenous antioxidant and its thiol (GSH) – disulphide (GSSG) redox couple is central to maintaining the redox environment of the cell. Glutathione (GSH) is glucoxidized to glutathione disulfide (GSSG) in a number of reactions [26]; its essential ROS biochemistry is shown in Figure 1. Changes in GSH concentration influence half-cell reduction potential and therefore the cell with higher GSH concentration is more resistant to oxidative stress. It has been shown that a change in this redox couple is correlated with the biological status of the cell such as proliferation, differentiation and apoptosis [20]. GSH is thus an excellent measure of oxidative stress *in vivo* [21]. Its levels are depleted with OS in diabetes, and it recovers readily when glucose control is exercised. The GSSG/2GSH couple has also been shown to play important role in modulating glucose homeostasis: GSH infusion in patients with impaired glucose tolerance potentiates β-cell response to glucose, while in diabetic patients it leads to an increase in body glucose disposal [22]. This effect of GSH is also seen in healthy non-diabetic subjects [23], thus emphasising the importance of GSH in regulating glucose metabolism. Glucose undoubtedly needs to be controlled in diabetic conditions since hyperglycemia is directly responsible for induction of ROS, however, glutathione levels also need to improve significantly since an optimal GSH concentration augments antioxidant defence and decreases susceptibility to ROS-induced damage.

We monitored newly diagnosed diabetic patients over a period of eight weeks during which they were treated with oral anti-diabetic drugs to control hyperglycemia. We monitored their fasting glucose, HbA_1C_, and GSH at 0, 4 and 8 weeks. We developed a mathematical model to study how GSH responds to glucose control, in order to identify pathophysiological differences between individuals on therapy.

## Materials and Methods

### Subjects

Newly diagnosed type 2 diabetic patients (n = 54) with a mean and standard deviation of the age being 48.11±10.32 attending the Diabetes Unit, KEM Hospital with fasting plasma glucose level >6.9 mmol/L and healthy non-diabetic subjects (n = 50) with a mean and standard deviation for the age being 33.08±11.76 with fasting plasma glucose levels ≤6.9 mmol/L were studied. Newly-diagnosed diabetic patients were defined as individuals who had blood glucose levels >6.9 mmol/L and HbA_1C_ values more than 6.5% (47.5 mmol/mol), had no diabetes associated secondary complications and were not on any anti-diabetic medication before the diagnosis. Non-diabetic subjects were volunteers from academic institutions in Pune. Additional details about the anthropomorphic characteristics, gender, age and BMI, and drug treatments for diabetic patients are provided in the Supplementary Information Section 1, Tables S1–S2 in File S1. Fasting blood samples were collected at baseline and 4 and 8 weeks later from diabetic patients and non-diabetic subjects. Diabetic patients were advised on diet and physical activity and were put on anti-diabetic drugs to control hyperglycemia as necessary. Patients were also advised not to take any oral antioxidant and multi-vitamin supplements. The following groups of subjects were excluded: pregnant women, individuals with excessive alcohol intake, chronic smokers and those receiving antioxidants, those with clinical infection and an inflammatory or malignant disease. Subjects with a recent cardiovascular event and symptomatic heart disease were also excluded. The study protocol was approved by the Institutional Ethical Committee, KEM Hospital and Research Centre, Pune, and written informed consent was obtained from all the individuals after the purpose and nature of the study had been explained.

### Sample preparation

Fasting blood samples were drawn from diabetic patients and non-diabetic subjects. Samples were centrifuged at 4000 rpm for 10 minutes to separate the plasma. The buffy coat was removed and the erythrocytes were washed three times in cold saline, and hemolyzed by adding ice-cold ultrapure water (MilliQ plus reagent grade; Millipore, Bedford, MA) to yield a 50% hemolysate. Aliquots of hemolysate were stored at −80°C until analysis. Plasma glucose was measured by GOD PAP (glucose oxidase Peroxidase) method on an autoanalyzer (Hitachi 902, Japan). HbA_1C_ was measured by using an HPLC cation exchange column on D10 HbA_1C_ analyzer (Bio-Rad Laboratories, Hercules, CA) in diabetic patients.

Reduced glutathione (GSH) from the erythrocyte hemolysate was estimated using 5, 5′-dithio-bis-2-nitrobenzoic acid (DTNB) according to the method of Akerboom and Sies [24]. Plasma insulin was measured using insulin kit (Mercodia, Uppsala, Sweden). Insulin resistance (HOMA-IR) and β-cell function (HOMA-β) were calculated using the fasting insulin and glucose concentrations using online calculator (Homeostatic Model Assessment) [25].

### Statistical analysis

Diabetic patients and non-diabetic subjects were divided into age groups: Above and below age 40. Multiple linear regression of GSH with respect to age and BMI in the two age groups is shown in the Supplementary Information Section 2, Tables S3–S4 in File S1. Regression analysis between fasting glucose and HbA_1C,_ carried out by pooling together diabetic patients and non-diabetic subjects (visits 1 and 3) values, resulted in the equation 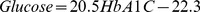 (see Supplementary Information, Section 7.1 and Figure S28 in File S1). A hierarchical clustering algorithm (Ward's algorithm) was used to evaluate a natural grouping in glutathione values, in order to categorize GSH ranges corresponding to non-diabetic subjects and diabetic patients (visits 1 and 3).

### Mathematical model and data fitting

The overall dynamics involving the interaction of GSH with glucose can be summarily represented as: 
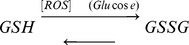



That is, GSH neutralizes ROS produced during glycolysis and converts to the disulfide form, GSSG. A *minimal* mathematical model is to assume the oxidation of GSH to GSSG (but not the reduction of GSSG to GSH) is proportional to plasma glucose, that is, to ROS. We modeled forward and backward reactions with saturation kinetics using Hill functions, thus: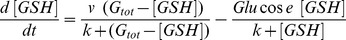,where 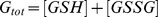 is the total glutathione in the two forms, 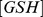 and 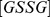. The physically relevant steady-state solution to this equation is the Goldbeter-Koshland function (see Supplementary Information, Section 5.1, for details).

We thus obtained a theoretical relation for the GSH response to serial changes in HbA_1C_ (converted to glucose values via the above relation). GSH and glucose measurements for each diabetic patient at 0, 4 and 8 weeks, together with a control value from non-diabetic subjects, were fit to the model. The control point from the non-diabetic data − this used to condition the asymptotic GSH in the fit − was obtained via a GSH versus age linear regression (Figure S14 in File S1) for each individual. Nelder-Mead optimization was used to obtain a best fit model in each case by minimizing least squares error. Further details of statistical analyses are followed in the Supplementary Information Section 5.2 in File S1.

## Results

### Beta-cell secretion and insulin sensitivity show improvement at 8 weeks

Metabolic imbalance challenges redox balance and impairs insulin sensitivity as well as insulin secretion, and glucose control ameliorates these conditions as measured through the homeostatic model assessment index, HOMA (see Figures S12 and S13; Figures S2–S9 in File S1 show insulin and glucose changes for each individual; Figures S10 and S11 in File S1 show average changes in glucose and insulin over 8 weeks, respectively).

### Depleted glutathione reserves recover rapidly over 8 weeks

**Figure 2:**
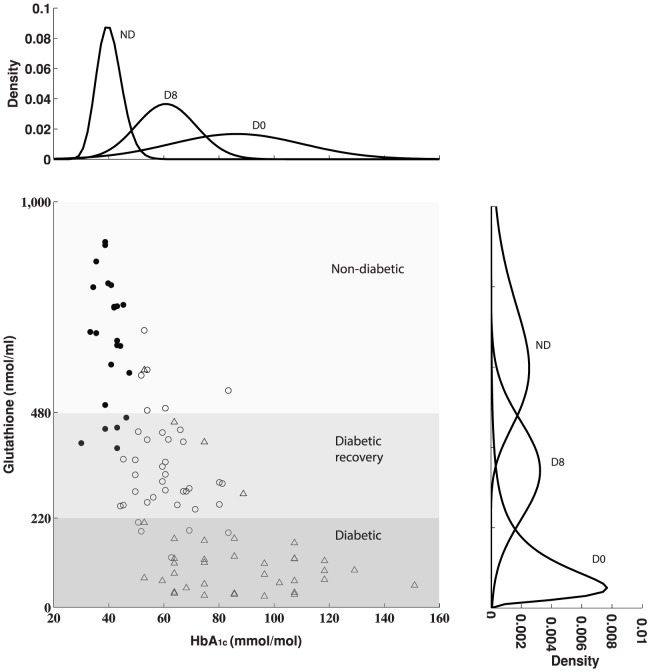
Reciprocal relationships between glycemia and the antioxidant GSH in recovering diabetic patients above age 40. ο: diabetic patients at 0 weeks (n = 38), Δ: diabetic patients at 8 weeks (n = 38), •: non-diabetic subjects at 0 and 8 weeks (n = 23). Three classes of the data are uncovered by cluster analysis: Diabetic patients have GSH less than 220, GSH between 220 and 480 represents a recovery phase at 8 weeks of treatment; non-diabetic subjects have GSH typically greater than 450. GSH of diabetic patients at 0 weeks was significantly different from that at 8 weeks (paired t-test, p<0.05). Probability density curves at the top and right of the figure are fitted distributions of HbA_1C_ and GSH respectively, of non-diabetic subjects (ND), and diabetic patients at 0 weeks (D0) and 8 weeks (D8). Data adapted from Acharya et al.^19^

Increases in glycolytic flux are expected to lead to increases in ROS via the TCA cycle, which are at least partially scavenged by glutathione together with other antioxidants; a decrease in glutathione concentration is therefore to be expected with hyperglycemia. To address whether GSH improves systematically in diabetic patients on an anti-diabetic regimen over two months, we correlated glutathione to HbA_1C_ of non-diabetic subjects and diabetic patients above age 40 at the beginning (0 week), at 4 weeks and at the end of study at 8 weeks (Figure 2). HbA_1C_ values of non-diabetic subjects lay largely between 4.9–6.5% (29.9–47.4 mmol/mol). Diabetic HbA_1C_ ranges between 7–16% (52.8–151 mmol/mol) with a mean and standard deviation of 10±2.2% (86.3±23.8 mmol/mol) before treatment and by 8 weeks of treatment is regulated to between 6.2–9.8% (44.1–83.3 mmol/mol) with mean and standard deviation, 7.7±1.0% (60.7±11.0 mmol/mol). Glutathione readings for non-diabetic subjects range between 392–900 nmoles/ml with a mean and standard deviation of 657±156 nmoles/ml. Mean and standard deviation values for glutathione for diabetics at 0-week and 8-weeks were 124±121 nmoles/ml and 342±123 nmoles/ml, respectively (see also Supplementary Information Section 3, Tables S5–S6 in File S1).

Population histograms (Figure 2) thus indicate a clear distinction between GSH values at 8 weeks of treatment relative to both, non-diabetic subjects and diabetic patients (above age 40) prior to treatment. To determine effective phase boundaries between these groups, glutathione data from both, diabetic patients and non-diabetic subjects at 0 and 8 weeks, was collectively pooled together and analyzed using a clustering algorithm. Glutathione values separate into groups partitioned at GSH = 220 and 480 nmoles/ml. Thus, the GSH ranges for the population are as follows: (i) >480 nmoles/ml: a healthy state for normoglycemia, (ii) 480–220 nmoles/ml: partial recovery at 8-weeks with glucose reduction following treatment and (iii) <220 nmoles/ml: a full-blown pathological state. The corresponding analysis for diabetic patients below age 40 is shown in Supplementary Information Section 3, Tables S7–S8, Figure S1 in File S1.

Our analysis demonstrates that in these diabetic patients GSH measurements can be used to reliably identify healthy persons from diabetic patients, and distinguish between patients before and after 8-weeks of treatment. While population measures show that, on average, glutathione increases in diabetic patients over 8-weeks, individual responses to glucose therapy vary considerably in both, the extent to which glucose is lowered as well as the degree to which GSH recovers with lowered glucose. We therefore developed a mathematical model to better understand the differences between the glucose-GSH responses of individuals.

### Modeling individual variation in the glutathione response to lowered glucose

**Figure 3:**
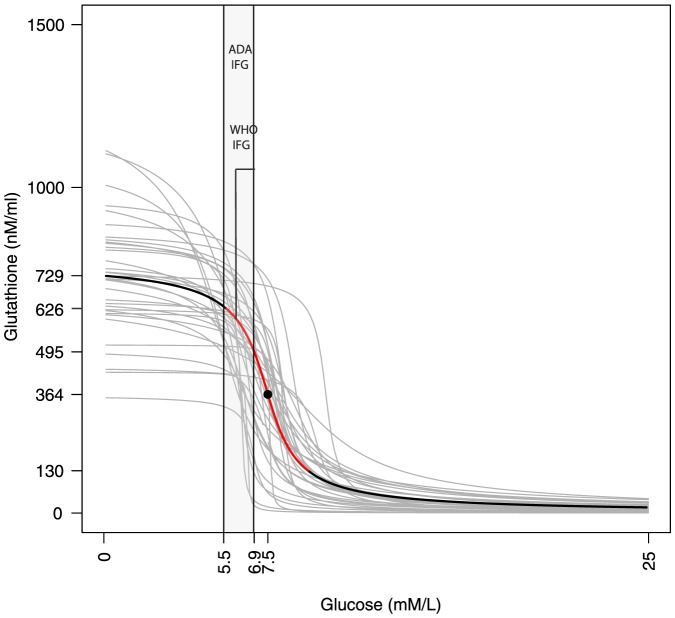
The GSH response to glucose reduction is unique to an individual patient. Individual response curves of diabetic patients obtained using the minimal model are shown (thin gray curves; n = 34, out of 49) together with an average profile (bold black curve). The population-averaged curve has a threshold (black dot) at glucose  = 7.5 mmol/L and GSH approximately 364; 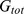 = 728, and 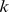  = 43.7. An inflection regime (width approximately one-fourth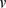, 1.8 mmol/L) is marked in red; this is a transition phase between fully developed hyperglycemia and normoglycemia. The ADA impaired fasting glucose (IFG) range, 5.5–6.9 mmol/L glucose, is overlaid for reference; WHO-IFG is the WHO criterion, 6.1–6.9 mmol/L. It is interesting to note that IFG occupies the upper third of the red curve, and 8-week patients (above age 40; GSH between 220–480 nmoles/ml) lie in the lower portion of the red curve.

We fit GSH and glucose data to the minimal model (see Materials and Methods, Mathematical Model) for each patient. Note that an age-dependence of GSH is implicitly accounted for in the fitting process, so that we fit patients in both age groups. In 34 out of 49 patients meaningful fits could be obtained (Figure 3) (see Supplementary Information, Section 5.2 for further details, Figures S15–S23 in File S1 for each the individual fits and Section 5.3, Figures S24–S25 in File S1 for the fits grouped into the two age groups, above and below 40).

The glutathione response to glucose is biphasic between levels corresponding to diabetic patients and non-diabetic subjects, and switches rapidly between them at a critical glucose threshold. Each fit is therefore characterized by three parameters: 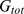 is the maximal GSH corresponding to very low glucose, 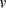 is the inflection point, i.e. the glucose concentration for which the glutathione is half-maximal, and 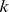 is a slope factor that determines the steepness of the glucose-GSH response curve. It is useful to think of 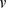 − in pharmacological “dose-response” terminology − as the EC_50_ of this GSH-glucose relationship.

Individual dose-response curves show considerable variation (Figure 3). Population mean and standard deviation values for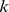, 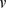 and 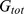
are 43.7±40, 7.5±1.1 and 728±178, respectively. Neither 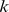 nor 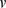 is significantly correlated with age or BMI. A “population-average curve” obtained by taking the mean
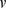
_,_ 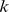 and 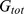
across diabetic patients (bold curve in Figure 3) reveals *quantal* characteristics of the average GSH response to glucose: Hyperglycemia is associated with a shallow low-glutathione phase; GSH rises sharply between glucose 5.6 and 9.3 mmol/L, and undulates to an asymptotic glutathione of about 728 nmoles/ml for normoglycemia.

The pathophysiological parameters
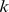
_,_ 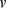 and 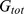
distinguish an individual's response to therapy from the average behavior; how this information might potentially be used to tailor treatment is discussed below.

### Glutathione in relation to clinical measures of diabetes

We propose that glucose control in anti-diabetic therapy can be reinterpreted as an attempt to control oxidative stress and improve systemic redox profile. The population-average curve (Figure 3) displays features consistent with glucocentric measures of diabetes and pre-diabetes. Half-maximal GSH occurs for glucose  = 7.5 mmol/L; this is greater than the upper limit for the ADA and WHO criteria of impaired fasting glucose (IFG). In other words, GSH lower than 495 nmol/ml represents the diabetic state, while higher GSH values represent healthy and pre-diabetic stages. Approximately 1.8 mmol/L (that is, 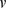 /4) on either side of the inflection point mark the most sensitive portion of the curve (in bold red) from the two relatively saturated phases at high or normal glucose (bold black). The GSH corresponding to the inflection region is 130 to 626. The ADA range for IFG occupies the upper third of this inflection region. In other words, IFG corresponds to GSH values that have deteriorated away from healthy levels and have just entered the inflection zone, but are nevertheless above half-maximal GSH.

Figure 3 also shows that 8 weeks is a period of substantial recovery in therapy for diabetic patients above age 40: 8-week GSH values have entered the inflection curve, albeit on the *lower* half (compare against Figure 2). 8-week GSH levels are thus just below the threshold of recovery.

We note that individuals respond differently from the population average. From the observations above we infer that when recovery is interpreted from a GSH viewpoint (see below as well, Implications for clinical therapy), a substantive recovery can be claimed to occur only once GSH has improved past its half-maximal threshold. This has implications for personalization of glucose control therapy; we discuss these next.

### Implications for clinical therapy

**Figure 4:**
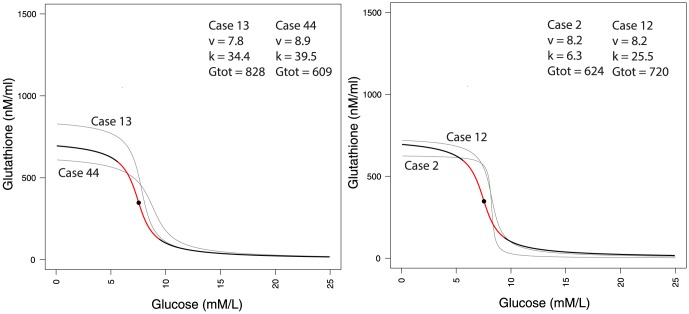
Glucose-GSH relationships for four individuals. In the two example cases in the left panel, the predicted thresholds of recovery are very different; in the right panel two patients have similar thresholds but very different slopes of recovery. Each response trajectory therefore merits personalization of the target of glucose control beyond eight weeks.

Although, overall, both insulin sensitivity and beta-cell secretion are expected to improve with lowered glucose, HOMA-IR measurements over 8 weeks cannot be used to evaluate the effectiveness of treatment for an individual. We hypothesize that because GSH has a threshold dependence on glucose, monitoring GSH over eight weeks can be used to quantitatively determine to what extent treatment is succeeding, and to make informed clinical decisions for personalization, such as whether glucose control should be intensified in the following weeks or not. We demonstrate this by example: Figure 4 shows a comparison of the glucose-GSH profiles of 4 individuals (ages greater than 40). In Figure 4 (left panel) the individuals are similar in terms of the *rate* with which GSH improves with glucose reduction, however, the glucose thresholds at which GSH is half-maximal are considerably different. In Case 44 the glucose threshold is at 8.9 mmol/L; we claim, therefore, that antioxidant benefits would already be near maximal even if the glucose were reduced only somewhat below 9.0 mmol/L. On the other hand, Case 13, whose threshold is at 7.8 mmol/L, is unlikely to get much OS relief unless glucose is lowered much further. Note that in either case the target glucose predicted by the analysis is higher than the IFG level; thus, these results suggest that anti-diabetic treatment can afford to have more relaxed controls here than typically recommended. In Figure 4 (right panel) we observe another aspect of the GS-OS threshold: in either Case 2 or 12 the threshold glucose is nearly the same; however, the steepness with which the threshold is expressed is very different in the two cases. For Case 2 OS can become maximal very rapidly, at only a few mmol/L below 8.2; in Case 12, however, OS will continue to slowly improve for glucose reduction well below threshold.

## Discussion

Our results show that patients with newly diagnosed type 2 diabetes mellitus respond to anti-diabetic glucose control medication with improved GSH levels that increase over eight weeks. A minimal mathematical model of antioxidant action indicates these increases in GSH are *nonlinearly* related to glucose reduction and are characterized by a glucose *threshold*. The model therefore predicts that lowering glucose below threshold can be expected to result in a dramatic improvement in GSH levels. In 34 of 49 patients we were able to quantitatively model the trajectories of individual responses; in each of these cases we obtain estimates of maximal glutathione at low stress, a glucose threshold for half-maximal glutathione, and a rate at which recovery progresses.

We have concentrated on investigating OS remission during therapy because antioxidant defense is likely to be a primary protection mechanism underlying the clinical control of glucose. Major strengths of this study include the use of GSH to obtain a quantifiable, objective measure of recovery from GS. The model itself is robust (for a detailed discussion of the assumptions that underlie the model, its behaviour and various features see Supplementary Information, Sections 5–7 in File S1; robustness issues are discussed in Figures S26–S27, S29 in File S1), and hence it was possible to use it reliably to deconstruct pathophysiological differences between individuals. The mathematical model is deliberately kept minimal, for two reasons: to retain the essential antioxidant action of glutathione in the simplest, robust fashion, and to avoid over-fitting data. The modelling procedures require only three measurements over two months; it remains to be seen if more frequent measurements of GSH and glucose would improve model estimates. It will also be interesting to construct more elaborate network models, since much is known about glutathione biochemistry; however, complex models will, in all likelihood, have to be fundamentally consistent with the minimal model. Should OS profiling become mainstay in diabetes research, such models could shed further light on the intricacies of individual differences. It will also be necessary to better understand sources of variability in glutathione both across, and within individuals. Further, our findings suggest that serial studies longer than eight weeks need to be carried out as they can reveal important information regarding the extent to which it is possible to push glucose control.

The proposals in our study, albeit motivated by excellent theoretical considerations, are currently speculative. This is an important limitation of our study; one that we hope will be addressed by epidemiological studies in the future. The technique we present can potentially aid in easing some of the complexity of personal therapy design. We stress, however, that therapy design is a complex process [1], [2] that involves multiple considerations. In other words, oxidative stress profiling can only be one of the tools – however crucial – in the clinician's repertoire. It would be interesting to study further nuances of our technique; such as how might factors like duration of the disease IR [1] or ER stress [27] be incorporated into the analysis. Another aspect is accounting for antioxidant capacity being influenced by factors unrelated to diabetes, co-existing infections for example. Finally, we note that oxidative stress is related not only to glucose but also several other molecules that are linked to diabetes, lipids for instance. While we have not found a significant variation of GSH with BMI in this study, in principle such dependence could exist, perhaps in other population groups. Future research will determine the extent to which the current method will be found effective, and what modifications will follow. Nevertheless, our method of oxidative stress profiling is readily amenable to clinical practice in its current form.

## Supporting Information

File S1 **File includes Figures S1–S29 and Tables S1–S8.** Figure S1: Cluster analysis of GSH values pulled together from non-diabetics and diabetics 0 and 8 weeks, age group below 40. •: non-diabetics 0 and 8 weeks (n = 72), ○: diabetics 0 week (n = 11), △: diabetics 8 weeks (n = 11). Three clusters emerged from the cluster analysis. Unlike the cluster analysis for the age group above 40 as shown in the main text, Fig. 2, below 40 GSH values do not show separation within diabetics groups 0 and 8 weeks. However, the non-diabetic below-40 age group is separated into two clusters, which shows apparent within-group age dependence on GSH levels. Figures S2–S9: Serial changes in plasma insulin and glucose for diabetic cases 1–54. ▴: 0 week, △: 4 weeks and •: 8 weeks. Figure S10: Average change in plasma glucose levels in diabetics kept on the anti-diabetic treatment for 8 weeks (n = 46). Mean and standard deviation values of plasma glucose corresponding to 0, 4 and 8 weeks are 10.7±3.3, 8.3±2.3 and 7.6±1.7, respectively. Paired t-test of mean change in plasma glucose at 0 and 8 weeks shows statistical significance, with p-value <0.05 at a 95% confidence interval. Figure S11: Average change in plasma insulin levels in diabetics kept on the anti-diabetic treatment for 8 weeks (n = 46). Mean and standard deviation values of plasma insulin corresponding to 0, 4 and 8 weeks are 11.6±8.2, 11.6±7.3 and 12.0±8.7, respectively. Though, there is slight increase in insulin secretion over 8 weeks, paired t-test of mean change in plasma insulin at 0 week and 8 weeks is not statistically significant, with p-value = 0.7 at a 95% confidence interval. Figure S12: HOMA2-IR against Glucose for non-diabetics and diabetics at 0 and 8 weeks. The bold line indicates serially observed change in the diabetics while the dotted line shows is a projection that assumes that if diabetics were to continue on the therapy for longer time period the asymptotic values of HOMA2-IR may lie close to the non-diabetic numbers. Figure S13: HOMA2-%B against GSH for non-diabetics and diabetics at 0 and 8 weeks. As in the previous figure, the bold line indicates serially observed change in the diabetics and the dotted line shows is a projection that assumes that if diabetics were to continue on the therapy for longer time period the asymptotic values of HOMA2-%B may lie close to the non-diabetic numbers. Figure S14: Linear regression of GSH against age in non-diabetics (n = 48). GSH levels are affected due to aging in non-diabetics. The equation for this regression line is GSH  = 1354.5–14.3×age, where p-values for the intercept and slope being <0.05 and 0.0002, respectively, at a 95% confidence interval. BMI doesn't contribute to GSH levels significantly (Data not shown, p-value for the slope of −5.24 being 0.73 at a 95% confidence interval). Figures S15–S23: Individual sigmoid fits for diabetic cases 1–54. In each case the diabetic patient's glucose and GSH pair at 0 week (□), 4 weeks (○) and 8 weeks (△) are shown alongside glucose-GSH pair taken from non-diabetic subjects from their first visit (▴) using regression fit. The pathophysiological parameters v, k and Gtot estimated from a fit are displayed in its panel. Figure S24: Individual response curves for diabetic patients above age 40 obtained using the minimal model are shown (thin gray lines, n = 29 out of 38) along with population-averaged curve (black bold line). The population-averaged curve has a threshold (black dot) at glucose  = 7.5 mmol/L and GSH approximately 347; Gtot = 695 and k = 43. An inflection regime is marked in red (width approximately one fourth of v, 1.87 mmol/L) is marked in red. The ADA impaired fasting glucose (IFG) range, 5.5–6.9 mmol/L and WHO IFG range 6–6.9 mmol/L is overlaid for the reference. The GSH band at 220–480 is the recovery phase for treated diabetics as shown in the main text, Figure 2. It is interesting to note that IFG occupies upper portion of the red curve, and 8-weeks patients lie in the lower portion of the red curve. Figure S25: Individual response curves for diabetic patients below age 40 obtained using the minimal model are shown (thin gray lines, n = 5 out of 11) along with population-averaged curve (black bold line). The population-averaged curve has a threshold (black dot) at glucose  = 7.4 mmol/L and GSH approximately 462; Gtot = 924 and k = 48.7. An inflection regime is marked in red (width approximately one fourth of v, 1.85 mmol/L) is marked in red. The ADA impaired fasting glucose (IFG) range, 5.5–6.9 mmol/L and WHO IFG range 6–6.9 mmol/L is overlaid for the reference. Unlike the above 40 group, cluster analysis does not show GSH separation for diabetics recovery as shown in the figure S1. Nonetheless, the IFG band lies in the sensitive upper portion of the red curve. Figure S26: A comparison of GSH values of non-diabetic subjects and theoretical predictions of GSH values of diabetic patients at glucose were 5.2 mmol/L. This plot shows the natural variability in GSH at low glucose, in non-diabetics and diabetics. Figure S27: Distributions of v, k and Gtot in the diabetics above age 40. Mean and standard deviation values for v, k and Gtot are 7.5±1.1, 43.0±40.0 and 695±166, respectively. Figure S28: Linear regression between fasting glucose and HbA1c. Fasting glucose and Hb1Ac values were taken from •: Non-diabetic; °: diabetic 0 week; □: diabetic 4 weeks; △: diabetic 8 weeks. This equation is used to convert HbA1c into a glucose value for model fitting. Figure S29: Distributions of the parameters v, k and Gtot for the samples cases 15 and 13. Table S1: Summary of anthropomorphic characteristics: Gender, age and BMI of non-diabetics (n = 48) and diabetics (n = 49) used in the data analysis. Table S2: Summary of anti-diabetic drug treatment given to 48 diabetic subjects over the period of 8 weeks. Out of 48 diabetics, 58% received DPP-4 inhibitor or gliptin treatment, 21% received biguanide drug treatment and remaining 21% received combination of biguanides and sulphonamides drug treatment. Table S3: Multiple linear regression of 0-week GSH with Age and BMI, in non-diabetics (n = 12) and diabetics (n = 38) above age 40. Both age and BMI are not significant predictors of GSH within non-diabetic and diabetic groups. Table S4: Multiple linear regression of 0-week GSH with age and BMI, in non-diabetics (n = 36) and diabetics (n = 11) below age 40. In both groups BMI is not significant predictor of GSH. However, age predicts GSH in non-diabetics, but not in diabetics. Table S5: Mean and standard deviation values corresponding to normal or log-normal probability density curves fitted to GSH and HbA1c levels of non-diabetics and diabetics shown in the Figure 1 in the main text. Table S6: A hierarchical cluster analysis performed on GSH values of non-diabetics (n = 23), diabetics at 0 week (n = 38) and diabetics at 8 weeks (n = 38) showed 3 clusters emerging from the data. For example, cluster 1 comprises of 3 diabetics at 0 week, 27 diabetics from 8 weeks and 5 non-diabetic. Based on this information we could distinguish between diabetics, before and after treatment, and non-diabetics, as shown in the Figure 2 in the main text. Table S7: Mean and standard deviation values corresponding to normal or log-normal probability density curves fitted to GSH and HbA1c levels of non-diabetics and diabetics below age 40. Table S8: A hierarchical cluster analysis performed on GSH values of non-diabetics (n = 72) 0 and 8 weeks together, diabetics at 0 week (n = 11) and diabetics at 8 weeks (n = 11) showed 3 clusters emerging from the data.
(PDF)

## References

[1] American Diabetes Association (2013) Standards of medical care in diabetes-2013. Diabetes Care 36 (suppl 1)S11–66. 23264422 10.2337/dc13-S011 PMC3537269

[2] Raz I, Riddle MC, Rosenstock J, Buse JB, Inzucchi SE, et al (2013) Personalized management of hyperglycemia in type 2 diabetes: reflections from a Diabetes Care Editors' Expert Forum. Diabetes Care 36(6): 1779–88. 23704680 10.2337/dc13-0512 PMC3661796

[3] Abdul-Ghani MA, Matsuda M, Subbah M, Jenkison CP, Richardson DK, et al (2007) The relative contributions of insulin resistance and beta cell failure to the transition from normal to impaired glucose tolerance varies in different ethnic groups. Diabetes Metab Syndr 1: 105–112.

[4] Evans JL, Goldfine ID, Maddux BA, Grodsky GM (2002) Oxidative stress and stress activated signaling pathways: a unifying hypothesis of type 2 diabetes. Endocr Rev 23(5): 599–622. 12372842 10.1210/er.2001-0039

[5] Houstis N, Rosen ED, Lander ES (2006) Reactive oxygen species have a causal role in multiple forms of insulin resistance. Nature 440: 944–48. 16612386 10.1038/nature04634

[6] Hoehn KL, Salmon AB, Hohnen-Behrens C, Turner N, Hoy AJ, et al (2009) Insulin resistance is a cellular defense mechanism. Proc Natl Acad Sci USA 106(42): 17787–92. 19805130 10.1073/pnas.0902380106 PMC2764908

[7] Evans JL, Maddux BA, Goldfine ID (2005) The molecular basis for oxidative stress induced insulin resistances. Antioxid. Redox Signal. 7: 1040–1052. 10.1089/ars.2005.7.1040 15998259

[8] Anderson EJ, Lustig ME, Boyle KE, Woodlief TL, Kane DA, et al (2009) Mitochondrial H2O2 emission and cellular redox state link excess fat intake to insulin resistance in both rodents and humans. J. Clin. Invest. 119: 573–581. 19188683 10.1172/JCI37048 PMC2648700

[9] Chen L, Na R, Gu M, Salmon AB, Liu Y, et al (2008) Reduction of mitochondrial H2O2 by overexpressing peroxiredoxin 3 improves glucose tolerance in mice. Aging Cell 7: 866–878. 18778410 10.1111/j.1474-9726.2008.00432.x PMC4431549

[10] Lee HY, Choi CS, Birkenfeld AL, Alves TC, Jornayvaz FR, et al (2010) Targeted expression of catalase to mitochondria prevents age-associated reductions in mitochondrial function and insulin resistance. Cell Metab. 12: 668–674. 21109199 10.1016/j.cmet.2010.11.004 PMC3013349

[11] Brownlee M (2001) Biochemistry and molecular cell biology of diabetic complications. Nature 414(6865): 813–820. 11742414 10.1038/414813a

[12] Meigs JB, Larson MG, Fox CS, Keaney JF Jr, Vasan RS, et al (2007) Association of oxidative stress, insulin resistance, and Diabetic risk phenotypes: the Framingham Offspring Study. Diabetes care 30(10): 2529–35. 17586736 10.2337/dc07-0817

[13] Paolisso G, Giugliano D, Pizza G, Gambardella A, Tesauro P, et al (1992) Glutathione infusion potentiates glucose-induced insulin secretion in aged patients with impaired glucose tolerance. Diabetes Care 15(1): 1–6. 10.2337/diacare.15.1.1 1737525

[14] Bensellam M, Laybutt DR, Jonas JC (2012) The molecular mechanisms of pancreatic β-cell glucotoxicity: Recent findings and future research directions. Mol Cell Endocrinol 364(1–2): 1–27. 22885162 10.1016/j.mce.2012.08.003

[15] Tajiri Y, Grill V (2000) Aminoguanidine exerts a β-cell function preserving effect in high-glucose cultured beta cells. International journal of Experimental Diabetes Research 1(2): 111–119. 11469395 10.1155/EDR.2000.111 PMC2477756

[16] Tanaka Y, Tran P, Harmon J, Robertson P (2002) A role for glutathione peroxidase in protecting pancreatic B-cells against oxidative stress in a model of glucose toxicity. Proc Natl Acad Sci USA 99: 12363–8. 12218186 10.1073/pnas.192445199 PMC129450

[17] Lortz S, Tiedge M (2003) Sequential inactivation of reactive oxygen species by combined overexpression of SOD isoforms and catalase in insulin-producing cells. Free Radic Biol Med 34: 683–8. 12633745 10.1016/s0891-5849(02)01371-0

[18] Wolf G, Aumann N, Michalska M, Bast A, Sonnemann J, et al (2010) Peroxiredoxin III protects pancreatic β-cells from apoptosis. Journal of Endocrinology 207(2): 163–175. 20807727 10.1677/JOE-09-0455

[19] Acharya JD, Pande AJ, Joshi SM, Yajnik CS, Ghaskadbi SS (2014) Treatment of hyperglycaemia in newly diagnosed diabetic patients is associated with a reduction in oxidative stress and improvement in β-cell function. Diabetes/Metabolism Research and Review (in press). 10.1002/dmrr.2526 24459082

[20] Jones DP, Carlson JL, Mody VC, Cai J, Lynn MJ, et al (2000) Redox state of glutathione in human plasma. Free Radic Biol Med 28(4): 625–35. 10719244 10.1016/s0891-5849(99)00275-0

[21] Schafer FQ, Buettner GR (2001) Redox environment of the cell as viewed through the redox state of the glutathione disulfide/glutathione couple. Free Radic Biol Med 30(11): 1191–212. 11368918 10.1016/s0891-5849(01)00480-4

[22] Paolisso G, Giugliano D, Pizza G, Gambardella A, Tesauro P, et al (1992) Glutathione infusion potentiates glucose-induced insulin secretion in aged patients with impaired glucose tolerance. Diabetes Care 15: 1–7. 10.2337/diacare.15.1.1 1737525

[23] Paolisso G, Di Maro G, Pizza G, D'Amore A, Sgambato S, et al (1992) Plasma GSH/GSSG affects glucose homeostasis in healthy subjects and non-insulin dependent diabetics. Am J Physiol 263: E435–40. 1415522 10.1152/ajpendo.1992.263.3.E435

[24] Akerboom TP, Sies H (1981) Assay of glutathione, glutathione disulfide and glutathione mixed disulfides in biological samples. Meth Enzymol 77: 373–82. 7329314 10.1016/s0076-6879(81)77050-2

[25] The Oxford Centre for Diabetes, Endocrinology and Metabolism, Diabetes Trials Unit. HOMA calculator. Available: http://www.dtu.ox.ac.uk. Accessed 2013 March 15.

[26] Meister A (1995) Glutathione metabolism. Meth Enzymol 251: 3–7. 7651209 10.1016/0076-6879(95)51106-7

[27] Eizirik DL, Cardozo AK, Cnop M (2008) The Role for Endoplasmic Reticulum Stress in Diabetes Mellitus. Endocr Rev 29(1): 42–61. 18048764 10.1210/er.2007-0015

