# Supplementary material for: Thresholds of Oxidative Stress in Newly Diagnosed Diabetic Patients on Intensive Glucose-Control Therapy
Source: PLoS One. 2014 Jun 27;9(6):e100897. doi: 10.1371/journal.pone.0100897 (PMC4074157; doi:10.1371/journal.pone.0100897)
Supplement: File S1 — File includes Figures S1–S29 and Tables S1–S8. Figure S1: Cluster analysis of GSH values pulled together from non-diabetics and diabetics 0 and 8 weeks, age group below 40. •: non-diabetics 0 and 8 weeks (n = 72), ○: diabetics 0 week (n = 11), △: diabetics 8 weeks (n = 11). Three clusters emerged from the cluster analysis. Unlike the cluster analysis for the age group above 40 as shown in the main text, Fig. 2, below 40 GSH values do not show separation within diabetics groups 0 and 8 weeks. However, the non-diabetic below-40 age group is separated into two clusters, which shows apparent within-group age dependence on GSH levels. Figures S2–S9: Serial changes in plasma insulin and glucose for diabetic cases 1–54. ▴: 0 week, △: 4 weeks and •: 8 weeks. Figure S10: Average change in plasma glucose levels in diabetics kept on the anti-diabetic treatment for 8 weeks (n = 46). Mean and standard deviation values of plasma glucose corresponding to 0, 4 and 8 weeks are 10.7±3.3, 8.3±2.3 and 7.6±1.7, respectively. Paired t-test of mean change in plasma glucose at 0 and 8 weeks shows statistical significance, with p-value <0.05 at a 95% confidence interval. Figure S11: Average change in plasma insulin levels in diabetics kept on the anti-diabetic treatment for 8 weeks (n = 46). Mean and standard deviation values of plasma insulin corresponding to 0, 4 and 8 weeks are 11.6±8.2, 11.6±7.3 and 12.0±8.7, respectively. Though, there is slight increase in insulin secretion over 8 weeks, paired t-test of mean change in plasma insulin at 0 week and 8 weeks is not statistically significant, with p-value = 0.7 at a 95% confidence interval. Figure S12: HOMA2-IR against Glucose for non-diabetics and diabetics at 0 and 8 weeks. The bold line indicates serially observed change in the diabetics while the dotted line shows is a projection that assumes that if diabetics were to continue on the therapy for longer time period the asymptotic values of HOMA2-IR may lie close to the non-diabetic num [file pone.0100897.s001.pdf]

Supplementary information: Thresholds of oxidative stress in newly diagnosed diabetics patients on intensive glucose-control therapy

Rashmi Kulkarni, Jhankar Acharya, Saroj Ghaskadbi, Pranay Goel

May 17, 2014

Contents

1 Anthropomorphic characteristics of non-diabetic and diabetic subjects and anti-diabetic treatment details **2**

2 Multiple linear regression of GSH dependence on Age and BMI within above/below age 40 groups **3**

3 Statistical details pertaining to GSH cluster-histograms for age groups above and below 40 **4**
3.1 Additional statistical details pertaining to Figure 2 in the main text 4
3.2 Cluster analysis details of GSH values for diabetics and non-diabetics below age 40 4

4 Plasma insulin and glucose changes in each diabetic individual over 0, 4 and 8 weeks; and the corresponding HOMA calculations **7**
4.1 Calculation of HOMA1-IR and HOMA2-IR 7
4.2 Serial changes in fasting plasma insulin and fasting plasma glucose in diabetics over 8 weeks . 7
4.3 Details of the changes in HOMA2-IR and HOMA2-%B values in diabetics kept on glucose therapy compared to non-diabetics 18

5 Derivation of the mathematical model and details of the individual diabetic fits shown in Figure 3 in the main text **20**
5.1 Derivation of the steady-state solution of the minimal GSH-glucose model 20
5.2 Fitting individual diabetic data to the minimal model 21
5.3 Details of glucose-GSH response curves and population-averaged curves for diabetics above and below age 40 21
5.4 Explanation of the natural variation in the GSH values of non-diabetics using the minimal model 21

6 Distributions of the parameters v, k and G_{tot} in the diabetic population above age 40 **35**

7 Estimation of the robustness of fits to the minimal model **36**
7.1 Accounting for variation in glucose levels in an individual 36
7.2 Accounting for the daily variation in GSH levels 36

1 Anthropomorphic characteristics of non-diabetic and diabetic subjects and anti-diabetic treatment details

Table S1 shows anthropomorphic characteristics of non-diabetic and diabetic subjects. In non-diabetics 2 subjects were removed: Case 16 was removed due to missing data and case 14 developed diabetes during the study period. In diabetics, 5 subjects were removed due missing data points either in glucose or 8-week GSH (GSH) (Cases 5,8,53) or in BMI (Cases 42,48). Therefore, anthropomorphic characteristics of 48 non-diabetics and 49 diabetics are listed below and used in data analysis.

**Characteristic**		**Non-diabetic**	**Diabetic**
Gender	Female	23	22
	Male	25	27
Age	Mean ± Std. Dev.	32.8 ± 11.78	47.8 ± 10.5
	Range	22-64	29-76
BMI	Mean ± Std. Dev.	23.75 ± 3.2	26.0 ± 3.6
	Range	16.8-33.3	20.3-41.6

Table S1: Summary of anthropomorphic characteristics:Gender, age and BMI of non-diabetics (n=48) and diabetics (n=49) used in the data analysis.

Table S2 gives details about anti-diabetic medication given to diabetic subjects for 8 weeks. Out of 49 diabetic subjects, details of 48 subjects was available.

**Drug treatment**	**Number of diabetics**
DPP-4 inhibitor	28
Biguanide	10
Combination of drugs (Biguanide and sulphonamides)	10

Table S2: Summary of anti-diabetic drug treatment given to 48 diabetic subjects over the period of 8 weeks. Out of 48 diabetics, 58% received DPP-4 inhibitor or gliptin treatment, 21% received biguanide drug treatment and remaining 21% received combination of biguanides and sulphonamides drug treatment.

2 Multiple linear regression of GSH dependence on Age and BMI within above/below age 40 groups

The following tables S3and S4 show the GSH dependence on Age and BMI, after dividing non-diabetics and diabetics into two age groups, above and below 40 age group. We observed that within the above 40 age group, GSH is not influenced by age or BMI; this indicates that age and BMI can be removed as confounding factors while interpreting cluster analysis results. However, age-dependence is observed in non-diabetic subjects below age 40. This is also reflected in a cluster analysis of the below 40 group as followed in the figure S1.

**Non-diabetics**		
**Predictor variable**	**Coefficient**	**p-value**
Intercept	1021	0.122
BMI	10.47	0.58
Age	-12.5	0.18
**Diabetics**		
**Predictor variable**	**Coefficient**	**p-value**
Intercept	111.5	0.6
BMI	-3.76	0.62
Age	2.16	0.34

Table S3: Multiple linear regression of 0-week GSH with Age and BMI, in non-diabetics (n=12) and diabetics (n=38) above age 40. Both age and BMI are not significant predictors of GSH within non-diabetic and diabetic groups.

**Diabetics**		
**Predictor variable**	**Coefficient**	**p-value**
Intercept	111.5	0.6
BMI	-3.76	0.62
Age	2.16	0.34
**Non-diabetics**		
**Predictor variable**	**Coefficient**	**p-value**
Intercept	1779	0.001
BMI	18.3	0.29
Age	-47.1	0.009

Table S4: Multiple linear regression of 0-week GSH with age and BMI, in non-diabetics (n=36) and diabetics (n=11) below age 40. In both groups BMI is not significant predictor of GSH. However, age predicts GSH in non-diabetics, but not in diabetics.

3 Statistical details pertaining to GSH cluster-histograms for age groups above and below 40

3.1 Additional statistical details pertaining to Figure 2 in the main text

Figure 2 in the main text is a cluster histogram showing GSH values of non-diabetics and diabetics above age 40 at 0 and 8 weeks, against their HbA_{1c} values.

Table S5 shows the mean and standard deviations of GSH and HbA_{1c} of each of the populations, diabetic

	**Fitted distribution**	**Mean**	**Standard deviation**
**GSH (nmol/ml)**			
Non diabetic	Log-normal	620.9	157.6
Diabetic 0 week	Log-normal	121.0	108.9
Diabetic 8 weeks	Normal	342.4	123
HbA_{1c} **(mmol/mol)**			
Non diabetic	Log-normal	36.8	4
Diabetic 0 week	Normal	86.3	23.8
Diabetic 8 weeks	Normal	60.7	10.9

Table S5: Mean and standard deviation values corresponding to normal or log-normal probability density curves fitted to GSH and HbA_{1c} levels of non-diabetics and diabetics shown in the Figure 1 in the main text.

and non-diabetic, at 0 and 8 weeks. Each populations were fit either to a normal or log-normal distribution as indicated. The Shapiro-Wilk test for normality and q-q plots were used to confirm the normality. Parameters of the normal and log-normal distributions (mean and standard deviation) are obtained in MATLAB using the functions *normfit()* and *lognfit()*, respectively.

Table S6 shows statistics of a hierarchical cluster analysis performed on the GSH values non-diabetics and

	**Non-diabetics (0 and 8 weeks)**	**Diabetics (0 week)**	**Diabetics (8 weeks)**
Cluster 1	5	3	27
Cluster 2	0	34	5
Cluster 3	18	1	6

Table S6: A hierarchical cluster analysis performed on GSH values of non-diabetics (n=23) , diabetics at 0 week (n=38) and diabetics at 8 weeks (n=38) showed 3 clusters emerging from the data. For example, cluster 1 comprises of 3 diabetics at 0 week, 27 diabetics from 8 weeks and 5 non-diabetic. Based on this information we could distinguish between diabetics, before and after treatment, and non-diabetics, as shown in the Figure 2 in the main text.

diabetics at 0 and 8 weeks, pooled together. Euclidean distance measure was used to produce the distance matrix and Ward's method was used to perform hierarchical clustering. In cluster 1: 22% of the values correspond to diabetics at 0 week and 71% correspond to diabetics at 8 weeks and 8% non-diabetics. 13% of the GSH values from cluster 2 correspond to diabetics at 8 weeks, and 34% correspond to the diabetics at 0 week and no non-diabetics. Clusters 3 comprised of 78% non-diabetics, 3% diabetics at 0 week and 16% diabetics at 8 weeks.

3.2 Cluster analysis details of GSH values for diabetics and non-diabetics below age 40

Figure S1 in the text is a cluster histogram plotting GSH values of non-diabetics and diabetics below age 40 at 0 and 8 weeks, against their HbA_{1c} values. Table S7 shows the mean and standard deviations of GSH and HbA_{1c} of each of the populations, diabetic and non-diabetic, at 0 and 8 weeks, age below 40.

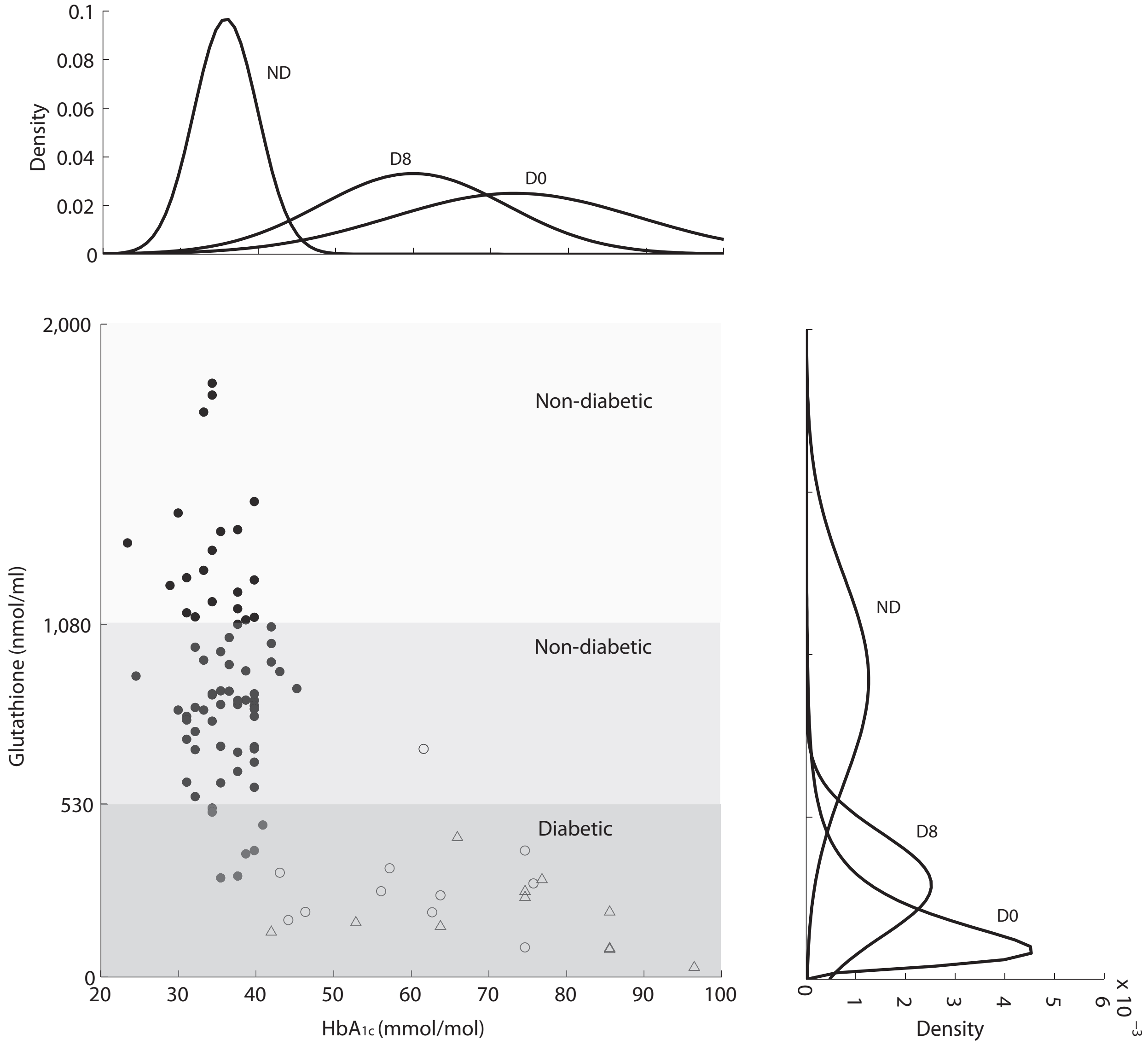

Figure S1: Cluster analysis of GSH values pulled together from non-diabetics and diabetics 0 and 8 weeks, age group below 40. • : non-diabetics 0 and 8 weeks (n=72), ○ : diabetics 0 week (n=11), △ : diabetics 8 weeks (n=11). Three clusters emerged from the cluster analysis. Unlike the cluster analysis for the age group above 40 as shown in the main text, Fig. 2, below 40 GSH values do not show separation within diabetics groups 0 and 8 weeks. However, non-diabetic below 40 age group is separated into two clusters, which shows apparent within group age dependence on GSH levels.

	**Fitted distribution**	**Mean**	**Standard deviation**
**GSH (nmol/ml)**			
Non diabetic	Normal	922.0	317.0
Diabetic 0 week	Log-normal	195.0	166.0
Diabetic 8 weeks	Normal	290.0	158.0
HbA_{1c} **(mmol/mol)**			
Non diabetic	Normal	35.8	4.1
Diabetic 0 week	Normal	73.0	15.9
Diabetic 8 weeks	Normal	60.0	12.0

Table S7: Mean and standard deviation values corresponding to normal or log-normal probability density curves fitted to GSH and HbA_{1c} levels of non-diabetics and diabetics below age 40.

Each populations were fit either to a normal or log-normal distribution as indicated. The Shapiro-Wilk test for normality and q-q plots were used to confirm the normality. Parameters of the normal and log-normal distributions (mean and standard deviation) are obtained in MATLAB using the functions *normfit()* and *lognfit()*, respectively.

Table S8 shows statistics of a hierarchical cluster analysis performed on the GSH values non-diabetics

	**Non-diabetics (0 and 8 weeks)**	**Diabetics (0 week)**	**Diabetics (8 weeks)**
Cluster 1	43	0	1
Cluster 2	22	0	0
Cluster 3	7	11	10

Table S8: A hierarchical cluster analysis performed on GSH values of non-diabetics (n=72) 0 and 8 weeks together, diabetics at 0 week (n=11) and diabetics at 8 weeks (n=11) showed 3 clusters emerging from the data.

and diabetics at 0 and 8 weeks, pooled together, below age 40. Euclidean distance measure was used to produce the distance matrix and Ward's method was used to perform hierarchical clustering. In cluster 1: 60% of the values correspond to non-diabetics, 9% correspond to 8-week diabetics and no 0-week diabetics. Cluster 2 corresponds to only non-diabetics which form 30% of the all non-diabetics, and cluster 3 contains 10% correspond to the 0-week diabetics and no non-diabetics. Clusters 3 comprised of 78% non-diabetics, all diabetics at 0 week and 91% diabetics at 8 weeks.

4 Plasma insulin and glucose changes in each diabetic individual over 0, 4 and 8 weeks; and the corresponding HOMA calculations

We sought to demonstrate that since oxidative stress is causal in the development of both beta-cell dysfunction and insulin resistance, glucose control does indeed ameliorate both conditions in the population.

We use the standard index, HOMA (Homeostatic Model Assessment), for evaluating insulin resistance and beta-cell function.

4.1 Calculation of HOMA1-IR and HOMA2-IR

Fasting plasma insulin (FPI) and fasting plasma glucose (FPG) values are used together in calculating HOMA. To estimate the insulin resistance in non-diabetics and diabetics, HOMA (now designated HOMA1-IR) was originally a simple formula that was a product of the two quantities [4] ,

$$HOMA = \frac{FPG \times FPI}{405}, \tag{1}$$

where FPG is expressed in mg/dL and FPI in mU/L.

A modified version of HOMA was proposed by Levy et al. [5]. An online HOMA2 calculator is available from http://www.dtu.ox.ac.uk/homacalculator/download.php. The two computations appear to give considerably different HOMA values for the same FPG and FPI values. For example, in the diabetic patient (Case 1, Figure S1), FPG (mg/dL) is 246 and FPI (mU/L) is 2.9, therefore using the product formula for this diabetic individual shows HOMA1-IR to be 1.76. HOMA2-IR requires FPG be expressed in terms of mmol/L and FPI is expressed as pmol/L. FPG (mg/dL) is converted to mmol/L using

$$FPG\ (mmol/L) = 0.055 \times FPG\ (mg/dL), \tag{2}$$

and FPI (mU/L) is converted to pmol/L using

$$FPI\ (pmol/L) = 6.945 \times FPI\ (mU/L). \tag{3}$$

Thus, for the diabetic patient considered above (Case 1, Figure S1), FPG in mmol/L is 13.53 and FPI in pmol/L is 20.14. These numbers are fed into the Excel sheet which calculates HOMA2-IR value using the HOMA2 model implemented as a macro. HOMA2-IR for this diabetic patient is 0.63, a value about three-fold smaller than HOMA1-IR.

Along with HOMA2-IR, the calculator simultaneously also computes an index, HOMA2-%B, to estimate β-cell function.

We use HOMA2-IR and HOMA2-%B for diabetics and non-diabetics (0 and 8 weeks) using the online HOMA2 calculator in our analysis.

4.2 Serial changes in fasting plasma insulin and fasting plasma glucose in diabetics over 8 weeks

Figures S2- S9 show individual diabetic plot of fasting plasma insulin against fasting plasma glucose values corresponding to $\blacktriangle$: 0 week, $\triangle$: 4 weeks and $\bullet$: 8 weeks. Altogether 54 patients were tested; of these, Cases 5, 8, 53 are not plotted due to missing glucose values and 17, 18, 31, 32, 43 are not plotted due to missing insulin values. Figures S10 and S11 show mean changes in glucose and insulin values in diabetics over the 8 weeks.

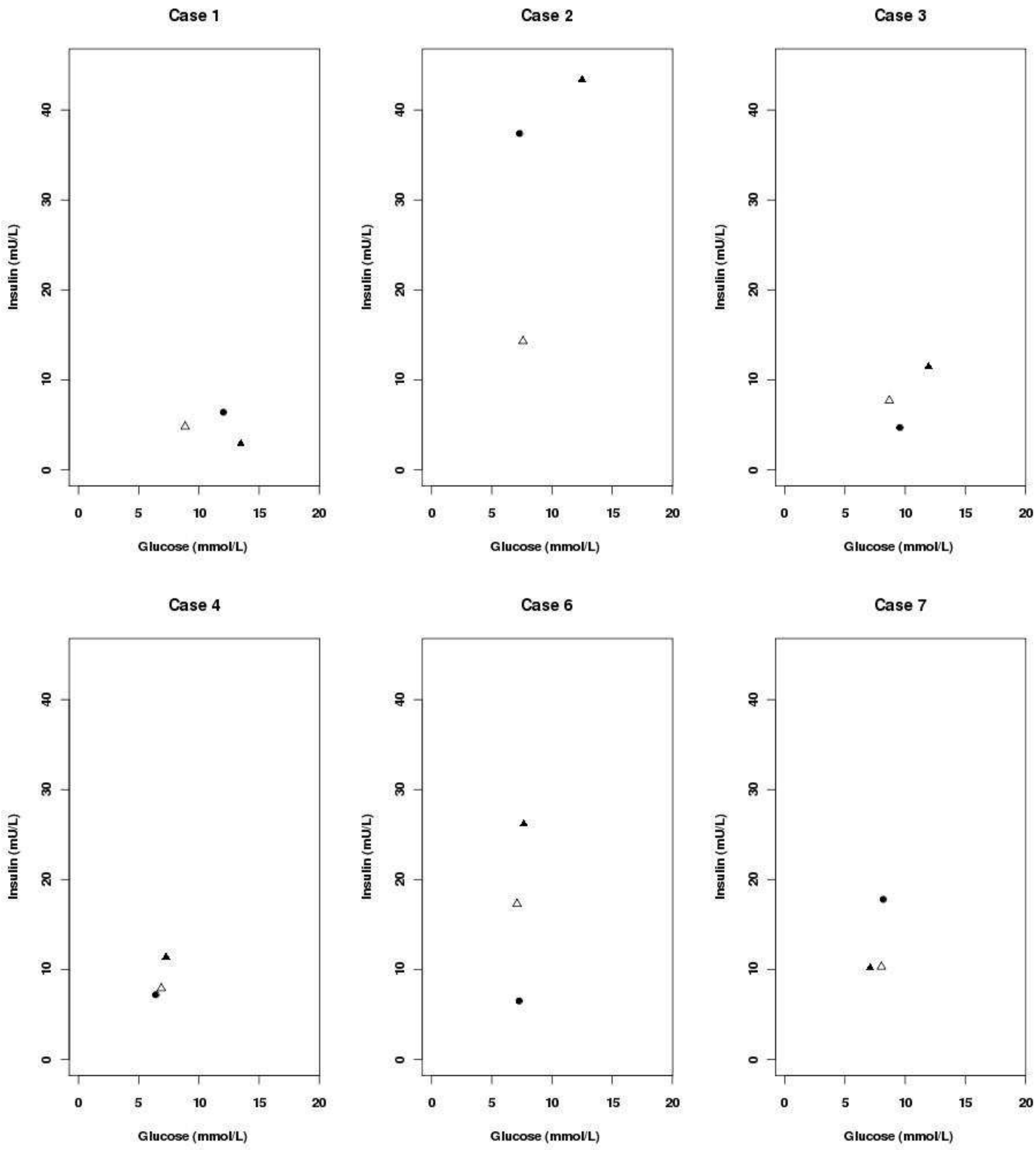

Figure S2: Serial changes in plasma insulin and glucose for diabetic cases 1-7. ▲ : 0 week, △ : 4 weeks and ● : 8 weeks.

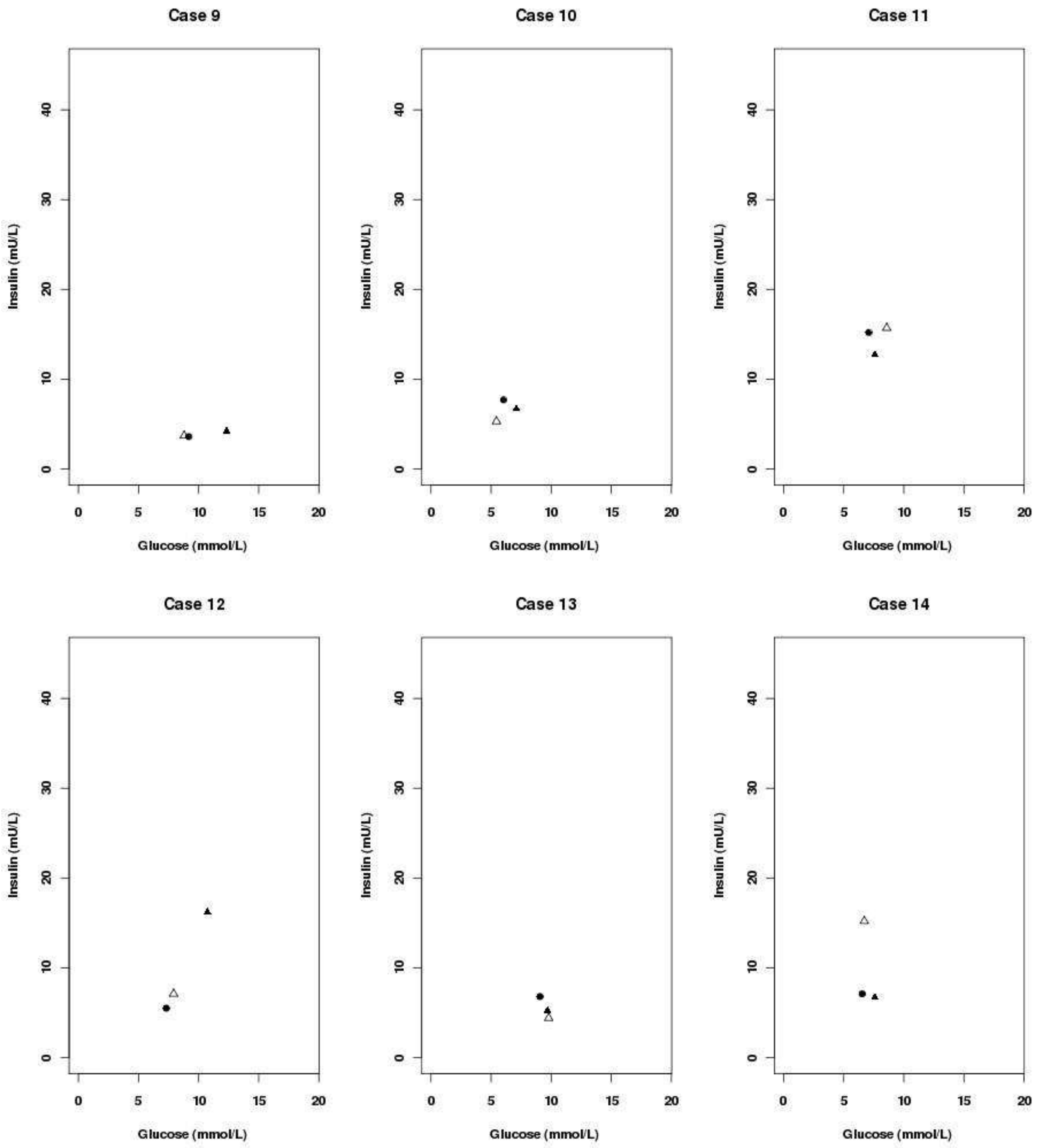

Figure S3: Serial changes in plasma insulin and glucose for diabetic cases 9-14. ▲ : 0 week, △ : 4 weeks and ● : 8 weeks.

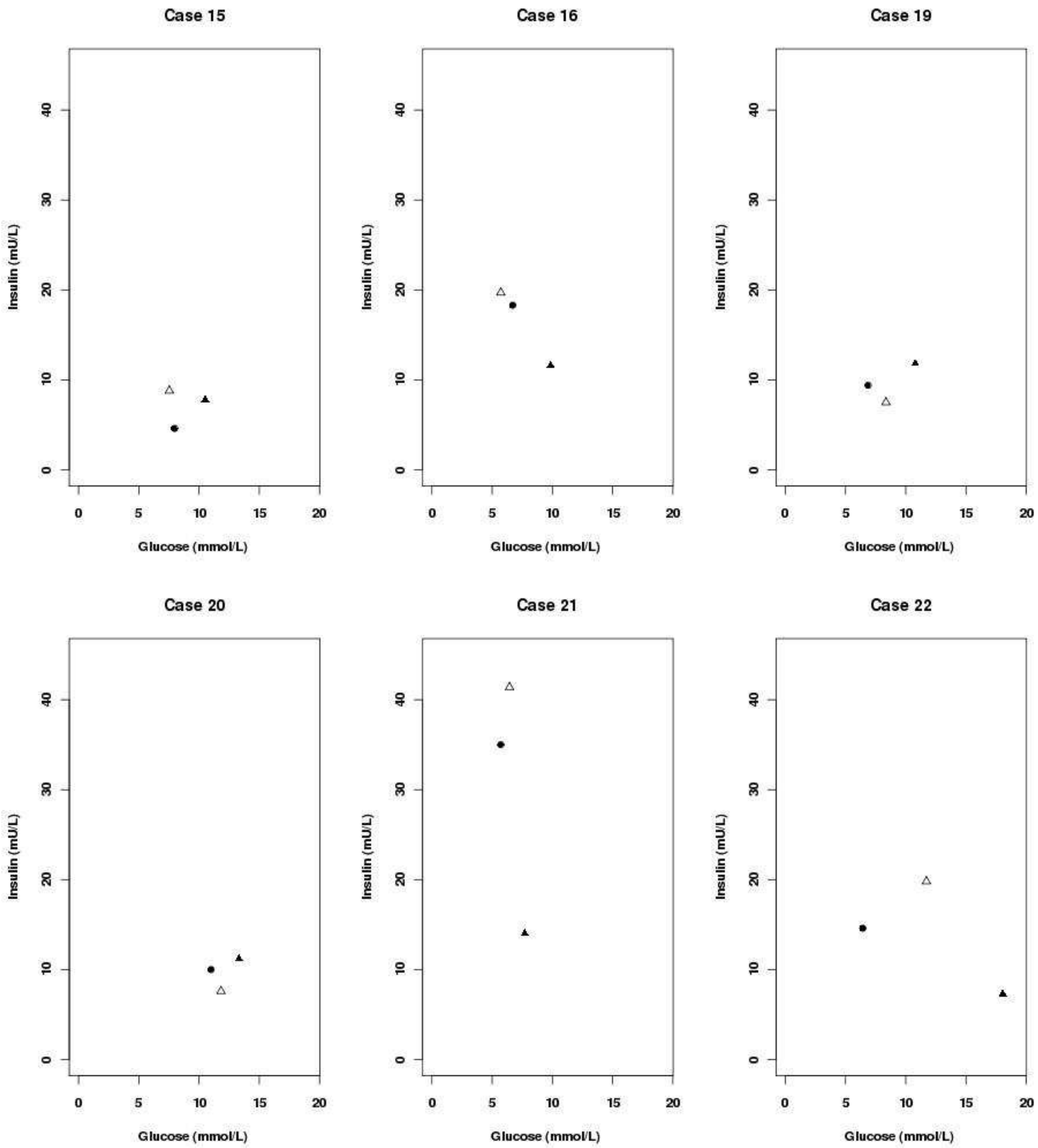

Figure S4: Serial changes in plasma insulin and glucose for diabetic cases 15-22. ▲ : 0 week, △ : 4 weeks and ● : 8 weeks.

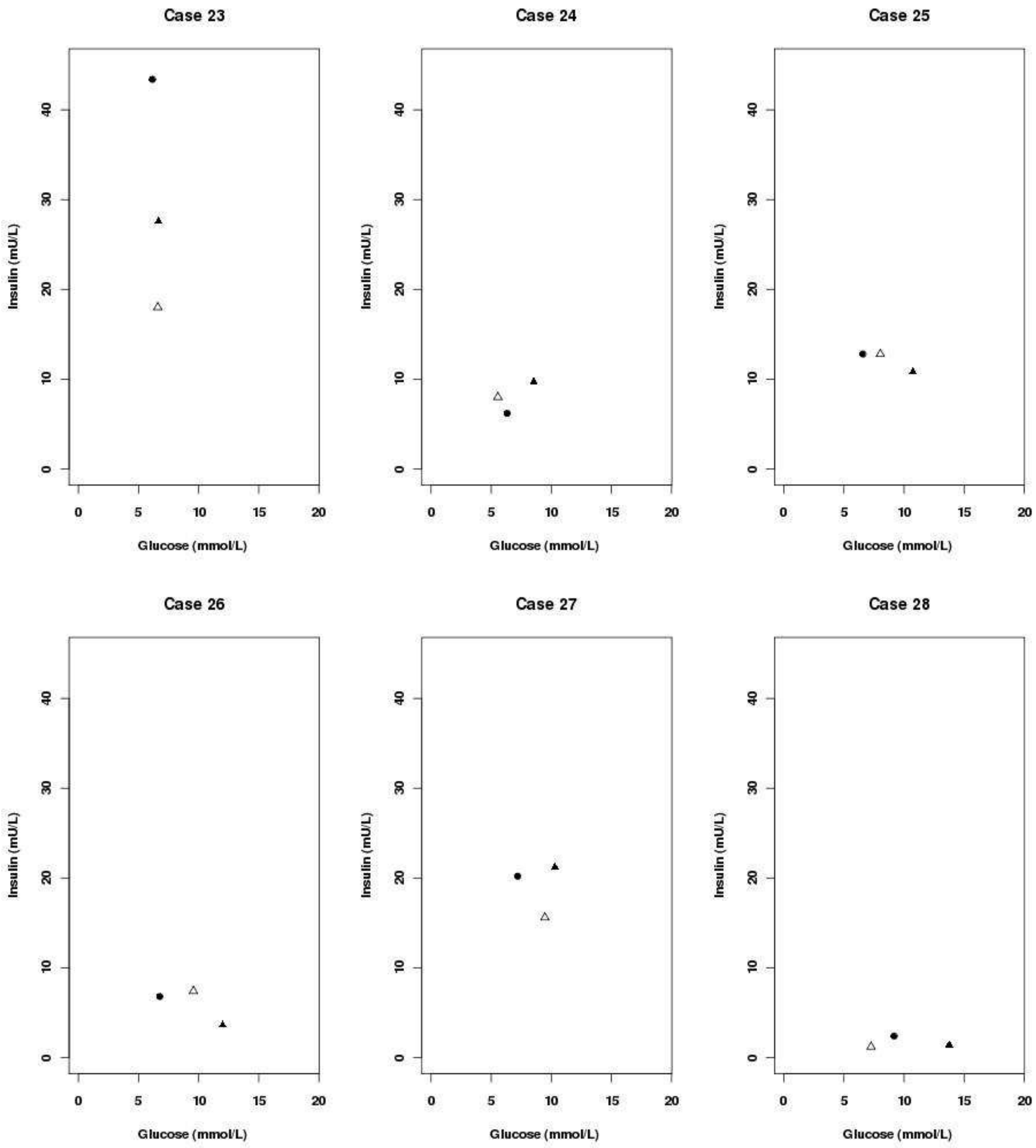

Figure S5: Serial changes in plasma insulin and glucose for diabetic cases 23-28. ▲ : 0 week, △ : 4 weeks and • : 8 weeks.

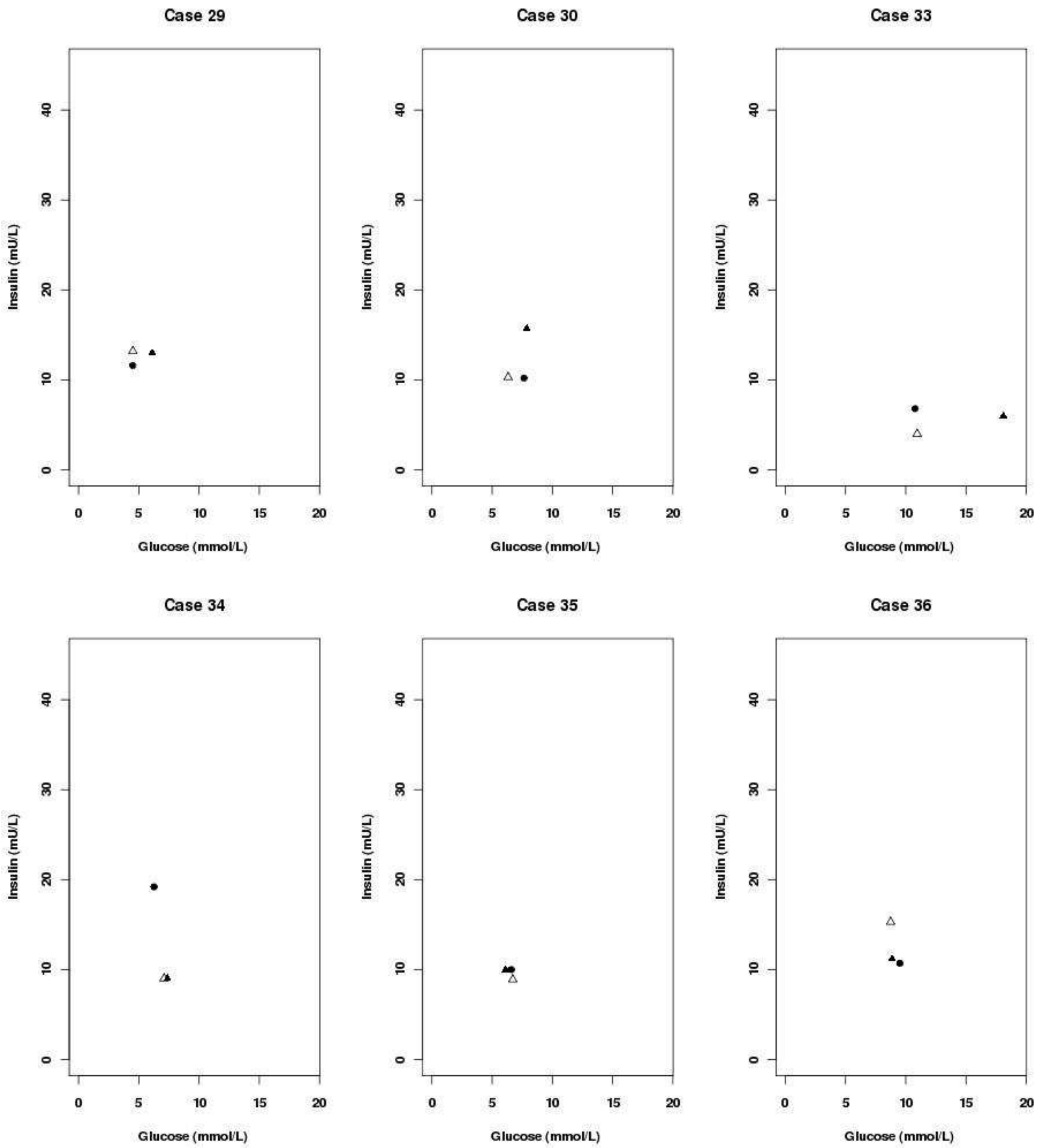

Figure S6: Serial changes in plasma insulin and glucose for diabetic cases 29-36. ▲ : 0 week, △ : 4 weeks and ● : 8 weeks.

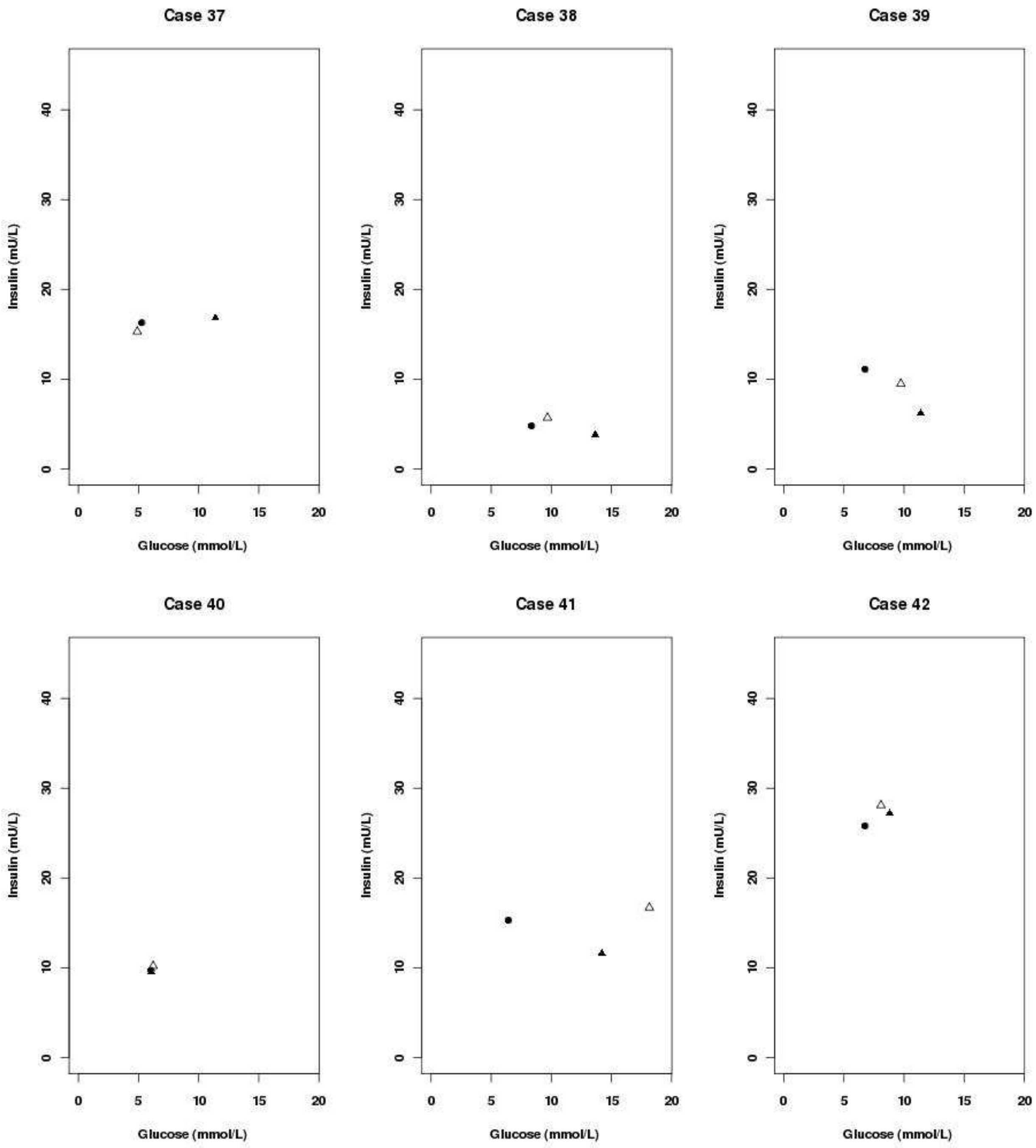

Figure S7: Serial changes in plasma insulin and glucose for diabetic cases 37-42. ▲ : 0 week, △ : 4 weeks and • : 8 weeks.

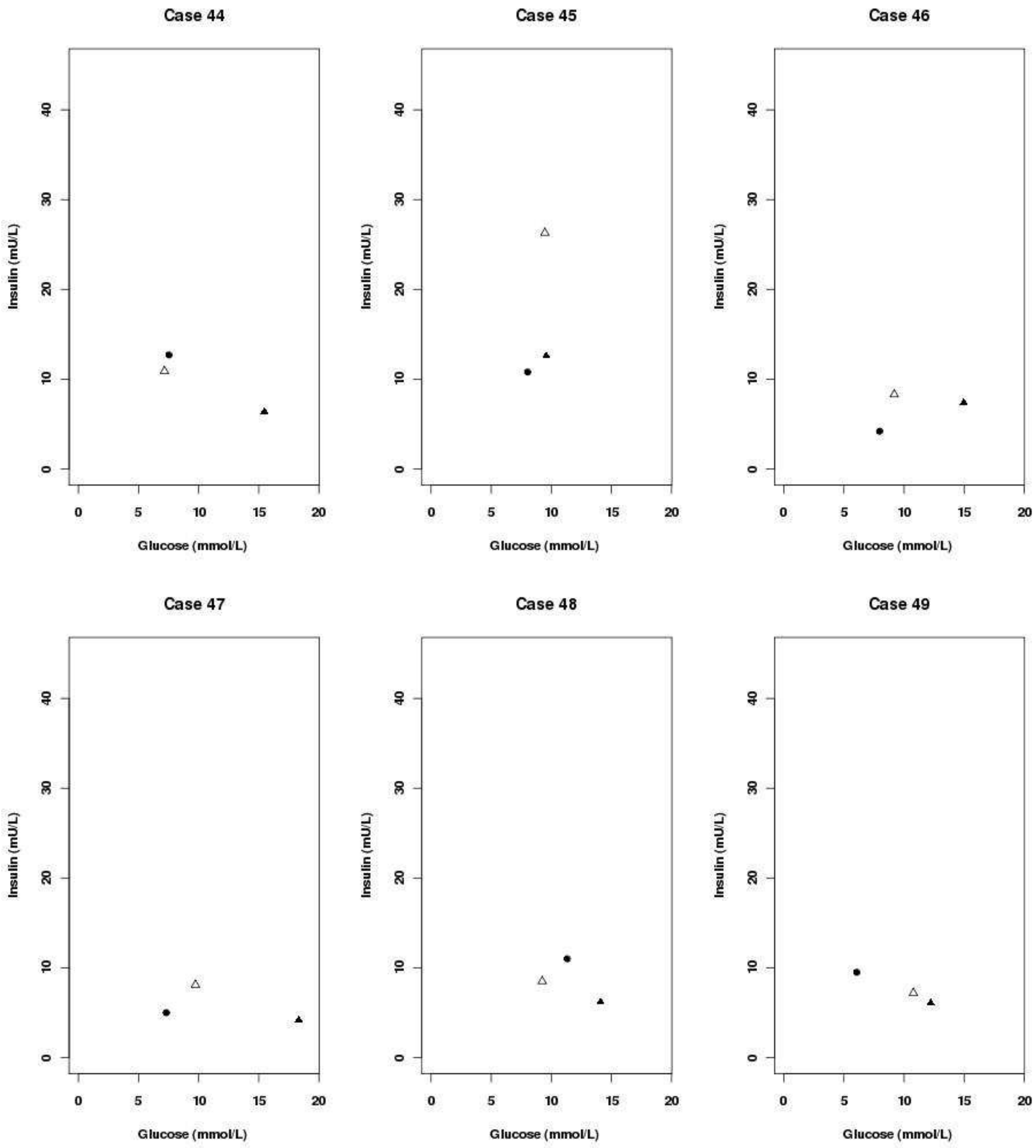

Figure S8: Serial changes in plasma insulin and glucose for diabetic cases 44-49. ▲ : 0 week, △ : 4 weeks and • : 8 weeks.

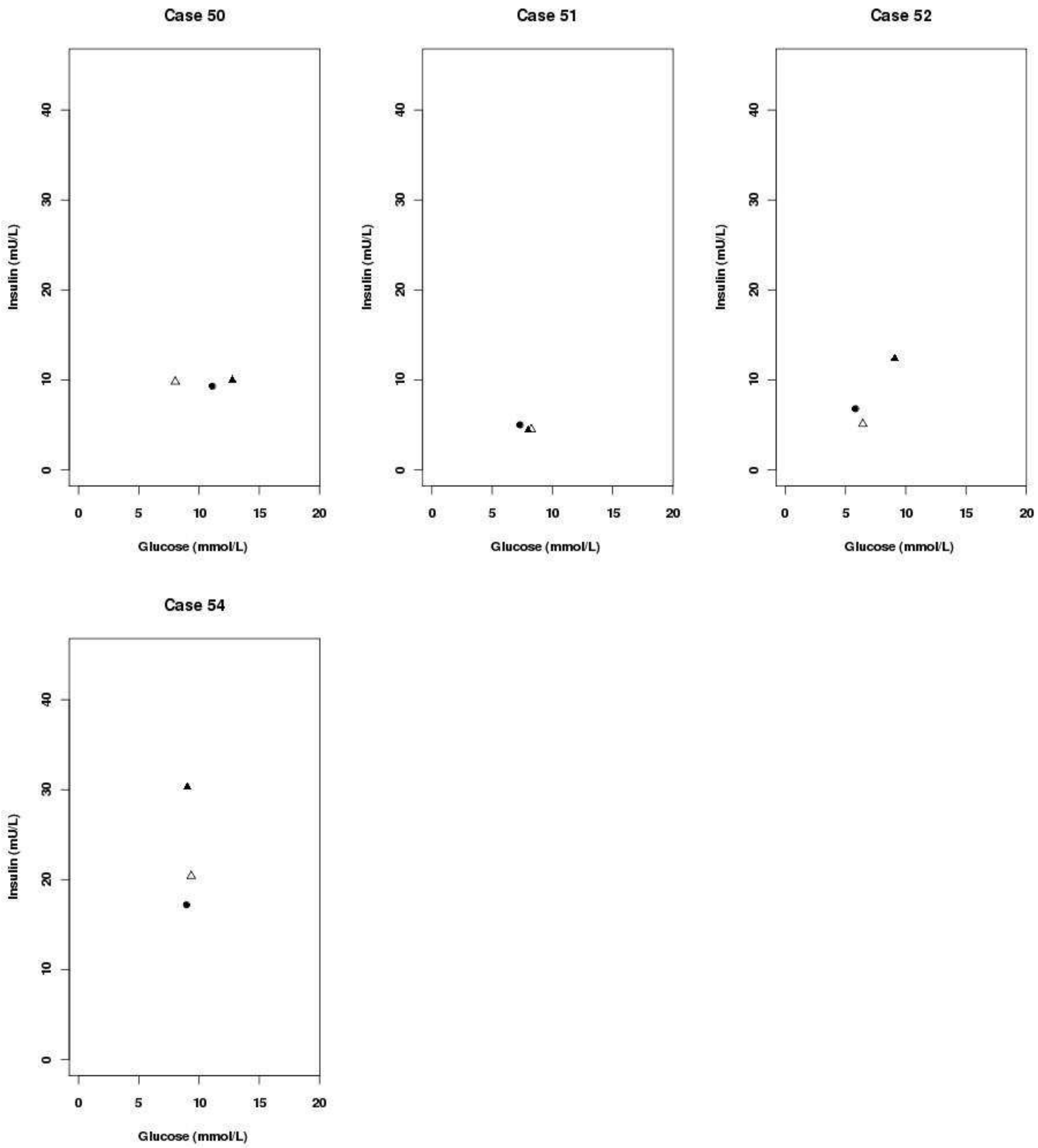

Figure S9: Serial changes in plasma insulin and glucose for diabetic cases 50-54. ▲ : 0 week, △ : 4 weeks and • : 8 weeks.

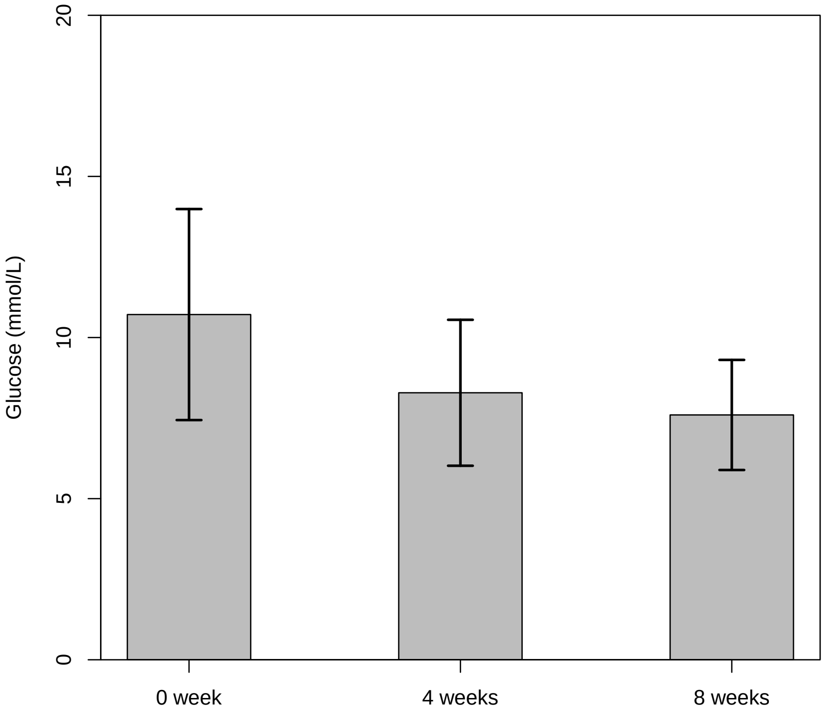

Figure S10: Average change in plasma glucose levels in diabetics kept on the anti-diabetic treatment for 8 weeks (n=46). Mean and standard deviation values of plasma glucose corresponding to 0, 4 and 8 weeks are 10.7 $\pm$ 3.3, 8.3 $\pm$ 2.3 and 7.6 $\pm$ 1.7, respectively. Paired t-test of mean change in plasma glucose at 0 and 8 weeks shows statistical significance, with p-value <0.05 at a 95% confidence interval.

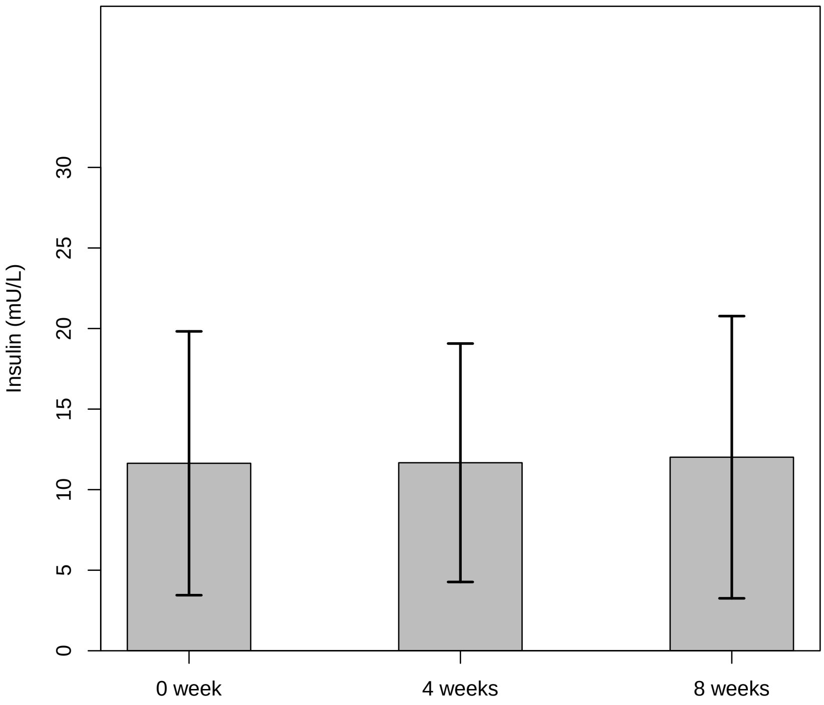

Figure S11: Average change in plasma insulin levels in diabetics kept on the anti-diabetic treatment for 8 weeks (n=46). Mean and standard deviation values of plasma insulin corresponding to 0, 4 and 8 weeks are 11.6 ± 8.2, 11.6 ± 7.3 and 12.0 ± 8.7, respectively. Though, there is slight increase in insulin secretion over 8 weeks, paired t-test of mean change in plasma insulin at 0 week and 8 weeks is not statistically significant, with p-value=0.7 at a 95% confidence interval.

4.3 Details of the changes in HOMA2-IR and HOMA2-%B values in diabetics kept on glucose therapy compared to non-diabetics

Figures S12 and S13 show improved mean insulin sensitivity and β-cell function as indicated by HOMA2-IR

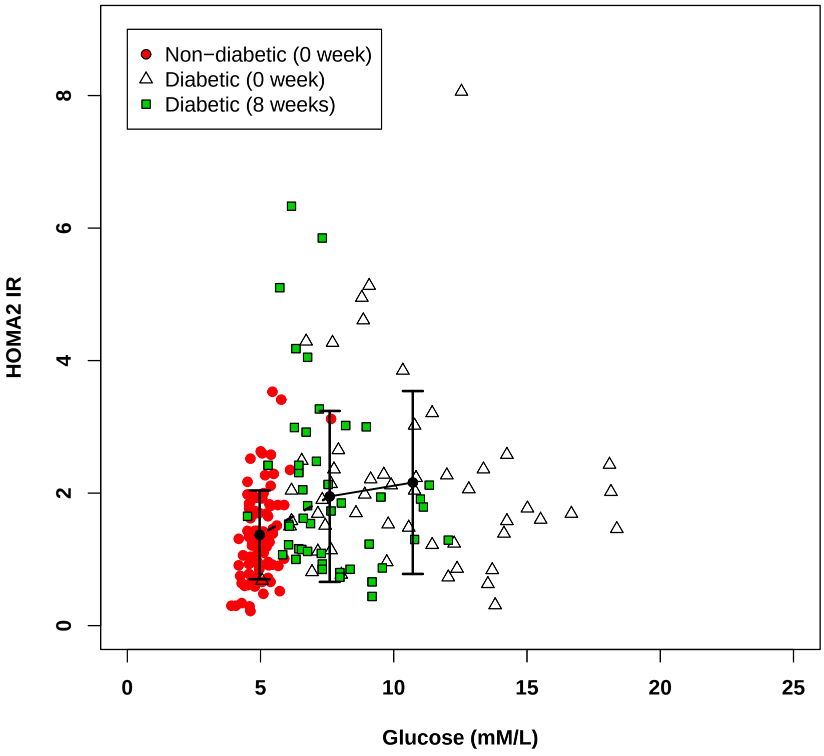

Figure S12: HOMA2-IR against Glucose for non-diabetics and diabetics at 0 and 8 weeks. The bold line indicates serially observed change in the diabetics while the dotted line shows is a projection that assumes that if diabetics were to continue on the therapy for longer time period the asymptotic values of HOMA2-IR may lie close to the non-diabetic numbers.

and HOMA2-%B scores in diabetics on the glucose control therapy for 8 weeks, respectively.

It should be noted, however, that because the variance in HOMA measures in each of the groups of non-diabetics (0 and 8 weeks) and diabetics (0 and 8 weeks) is large, the measurement of plasma insulin levels offer little additional help in assessing how glucose control influences insulin resistance or secretion intensively over the eight weeks period.

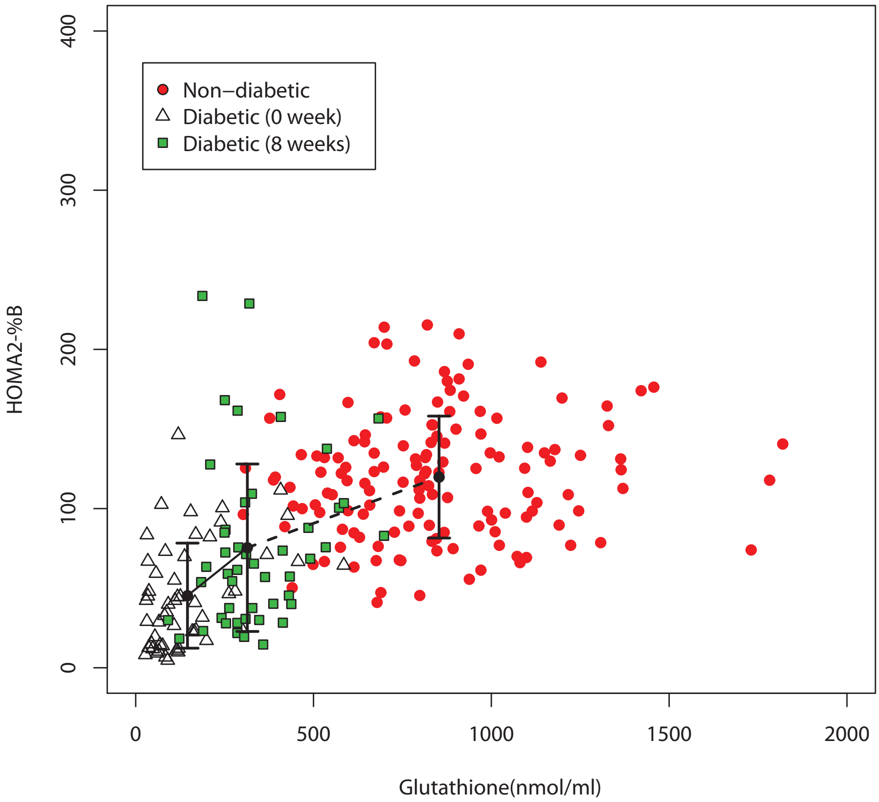

Figure S13: HOMA2-%B against GSH for non-diabetics and diabetics at 0 and 8 weeks. As in the previous figure, the bold line indicates serially observed change in the diabetics and the dotted line shows is a projection that assumes that if diabetics were to continue on the therapy for longer time period the asymptotic values of HOMA2-%B may lie close to the non-diabetic numbers.

5 Derivation of the mathematical model and details of the individual diabetic fits shown in Figure 3 in the main text

5.1 Derivation of the steady-state solution of the minimal GSH-glucose model

The minimal model for the antioxidant action of GSH, as described in the text, is to assume a ROS-dependent interconversion between the reduced (GSH) and oxidized form (GSSG). Taking the simplifying assumption that cellular ROS is roughly proportional to the blood glucose concentration, $[ROS] = \beta[Glucose]$, and using Michaelis-Menten kinetics for the forward and backward reactions, we have

$$\frac{d[GSH]_c}{d\tau} = \frac{v_1([G_{tot}]_c - [GSH]_c)}{k^\star + ([G_{tot}]_c - [GSH]_c)} - \frac{v_2\beta[Glucose][GSH]_c}{k^\star + [GSH]_c}, \tag{4}$$

where $[Glucose]$ is the plasma glucose concentration, $[G_{tot}]_c$ is total cellular GSH, $[GSH]_c$ is cellular $[GSH]$ and $[GSSG]_c = [G_{tot}]_c - [GSH]_c$ is cellular $[GSSG]$. Here we have taken the same constant, k, for both, forward and backwards reactions; this simplification is used to avoid overfitting the data. Introducing the rescaled variables $v = \frac{v_1}{v_2\beta}$ and $t = v_2\beta\tau$, we get

$$\frac{d[GSH]_c}{dt} = \frac{v([G_{tot}]_c - [GSH]_c)}{k^\star + ([G_{tot}]_c - [GSH]_c)} - \frac{[Glucose][GSH]_c}{k^\star + [GSH]_c}. \tag{5}$$

Notice that the equation Eq. (5) is in terms of cytosolic GSH variables. Clinically, however, the measurements are most readily collected from the blood. We thus have to transform cytosolic variables to plasma variables. Reed et al. [1] describe a detailed mathematical model of GSH metabolism; they show that, to first order, plasma GSH varies in proportion to cellular GSH, that is, $[GSH]_c \approx \alpha[GSH]_b$, where $[GSH]_b$ is blood GSH. Moreover, they demonstrate that this relationship is valid even as oxidative stress varies, and it is valid between diabetics and healthy persons, see Fig. 6 in [1]. Eq. (5) can therefore be written in terms of plasma GSH as

$$\alpha\frac{d[GSH]_b}{dt} = \frac{v([G_{tot}]_c/\alpha - [GSH]_b)}{k^\star/\alpha + ([G_{tot}]_c/\alpha - [GSH]_b)} - \frac{[Glucose][GSH]_b}{k^\star/\alpha + [GSH]_b}. \tag{6}$$

Thus the equation for $[GSH]_b$, in terms of the quantities $G_{tot} = [G_{tot}]_c/\alpha$ and $k = k^\star/\alpha$, is

$$\alpha\frac{d[GSH]_b}{dt} = \frac{v(G_{tot} - [GSH]_b)}{k + (G_{tot} - [GSH]_b)} - \frac{[Glucose][GSH]_b}{k + [GSH]_b}. \tag{7}$$

Setting

$$\frac{d[GSH]_b}{dt} = 0, \tag{8}$$

the steady state expression of $[GSH]_b$ as it varies with $[Glucose]$ is thus obtained as

$$\frac{v(G_{tot} - GSH)}{k + (G_{tot} - GSH)} - \frac{Glu \cdot GSH}{k + GSH} = 0 \tag{9}$$

where we have dropped the square brackets and subscript for simplicity and abbreviated $[Glucose]$ as Glu. Thus, we have the following implicit relation between GSH and Glu:

$$v(G_{tot} - GSH) \cdot (k + GSH) - Glu \cdot GSH \cdot (k + (G_{tot} - GSH)) = 0. \tag{10}$$

By rearranging the terms in Eq. (10) we get,

$$(Glu - v) \cdot GSH^2 + (v \cdot G_{tot} - v \cdot k - Glu \cdot k - G_{tot} \cdot Glu) \cdot GSH + k \cdot v \cdot G_{tot} = 0. \tag{11}$$

This is a quadratic equation that can be solved for GSH in terms of glucose: its physically relevant solution is the Golbeter-Koshland formula [2]:

$$GSH(Glu) = \frac{-(v \cdot G_{tot} - v \cdot k - Glu \cdot k - G_{tot} \cdot Glu) \pm \sqrt{\begin{matrix}(v \cdot G_{tot} - v \cdot k - Glu \cdot k - G_{tot} \cdot Glu)^2 \\ -4 \cdot k \cdot v \cdot G_{tot}(Glu - v)\end{matrix}}}{2 \cdot (Glu - v)} \tag{12}$$

Or, equivalently, expanding the square term inside the square root we get the following from:

$$GSH(Glu) = \frac{-(v \cdot G_{tot} - v \cdot k - Glu \cdot k - G_{tot} \cdot Glu) - \sqrt{\begin{array}{c} v^2 \cdot {G_{tot}}^2 + v^2 \cdot k^2 + k^2 \cdot Glu^2 \\ +Glu^2 \cdot {G_{tot}}^2 + 2 \cdot Glu^2 \cdot k \cdot G_{tot} \\ -2 \cdot {G_{tot}}^2 \cdot Glu \cdot v + 2 \cdot k^2 \cdot v \cdot Glu \\ +2 \cdot v^2 \cdot k \cdot G_{tot} - 4 \cdot Glu \cdot G_{tot} \cdot v \cdot k \end{array}}}{2 \cdot (Glu - v)}. \quad (13)$$

This is the GSH-Glucose minimal model that the data is fit to.

Note that the equation is parameterized by the three quantities, v, k and G_{tot}, that will vary from individual to another. We have the following physical interpretations of these parameters:

1. Notice that if we set $Glu = 0$ in Eq. (9), $GSH = G_{tot}$ satisfies the equation. That is, G_{tot} can be interpreted as the maximal value of GSH at low glucose.
2. Taking $Glu = v$ in Eq. (9) we find $GSH = G_{tot}/2$. Thus v is the threshold glucose value for which GSH is half-maximal.
3. Taking logarithms in Eq. (9) and differentiating, and taking the limits $GSH \to G_{tot}/2$ as $Glu \to v$, we find that

$$GSH'(Glu \to v) = -\frac{G_{tot}}{8v}\left(2 + \frac{G_{tot}}{k}\right) \quad (14)$$

where the derivative GSH' is with respect to Glu. For a given value of G_{tot}, the larger the k, the smaller is the slope of the $GSH(Gluc)$ curve at the inflection point. Thus k can be interpreted as a slope with which the inflection in expressed; in other words, the rate of recovery. A smaller k implies that the GSH becomes rapidly near-maximal as Glu crosses below the v threshold.

5.2 Fitting individual diabetic data to the minimal model

Three glucose-GSH pairs for each diabetic (Figures S11-S19) at 0 week (□), 4 weeks (○) and 8 weeks (△), along with 49 glucose-GSH pairs taken from non-diabetics from their first visit (▲) were used for fitting. Since, there is age dependent change in the GSH levels of non-diabetics as shown in the figure S14, this linear regression equation was used to obtain asymptotic GSH level of non-diabetic as a 4th point in the curve fitting. Briefly, the age of diabetic individual was used to obtain a maximum level of GSH that can be achieved, comparable to a non-diabetic individual of the same age. Average of first visit (0 week) of non-diabetics glucose values is taken as mean glucose for the fit, which is 4.8 mM/L. A nonlinear least squares optimization of the data was carried out with respect to sigmoid in the Eq. (13). We used the function *optim()* in the statistical software package, R, which implements the Nelder-Mead simplex search algorithm.

A total of 48 diabetics were tested for fitting. Cases 5, 8 and 53 could not be fitted due to missing data and cases 42 and 48 removed due to missing BMI value. Of the remaining 49 diabetics, we could fit 34 individuals, which have physiologically meaningful parameter values.

Figures S15-S23 show the results of all the 49 individual fits, and the physiological parameters, v, k, and G_{tot} of each diabetic case.

5.3 Details of glucose-GSH response curves and population-averaged curves for diabetics above and below age 40

Figures S24and S25 show individual response curves for diabetics above and below age 40, respectively, along with their population-averaged curves.

5.4 Explanation of the natural variation in the GSH values of non-diabetics using the minimal model

The fits from the model can be used to explain the variation that is seen in the GSH values of non-diabetics, Figure 1 in the main text. The model predicts that individuals, both diabetic and non-diabetic, have

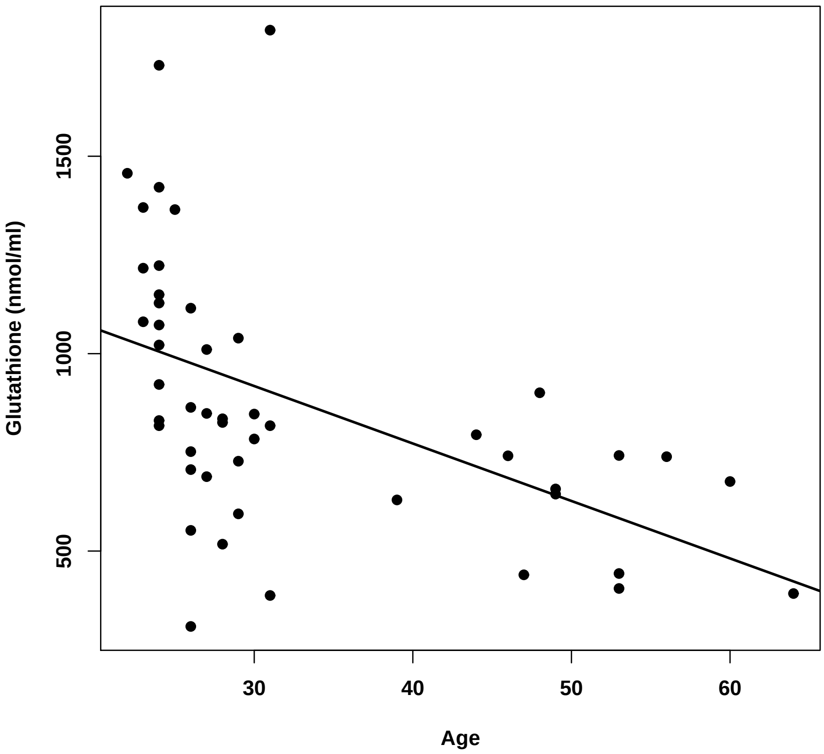

Figure S14: Linear regression of GSH against age in non-diabetics (n=48). GSH levels are affected due to aging in non-diabetics. The equation for this regression line is GSH = 1354.5 - 14.3 $\times$age, where p-values for the intercept and slope being <0.05 and 0.0002, respectively, at a 95% confidence interval. BMI doesn't contribute to GSH levels significantly (Data not shown, p-value for the slope of -5.24 being 0.73 at a 95% confidence interval).

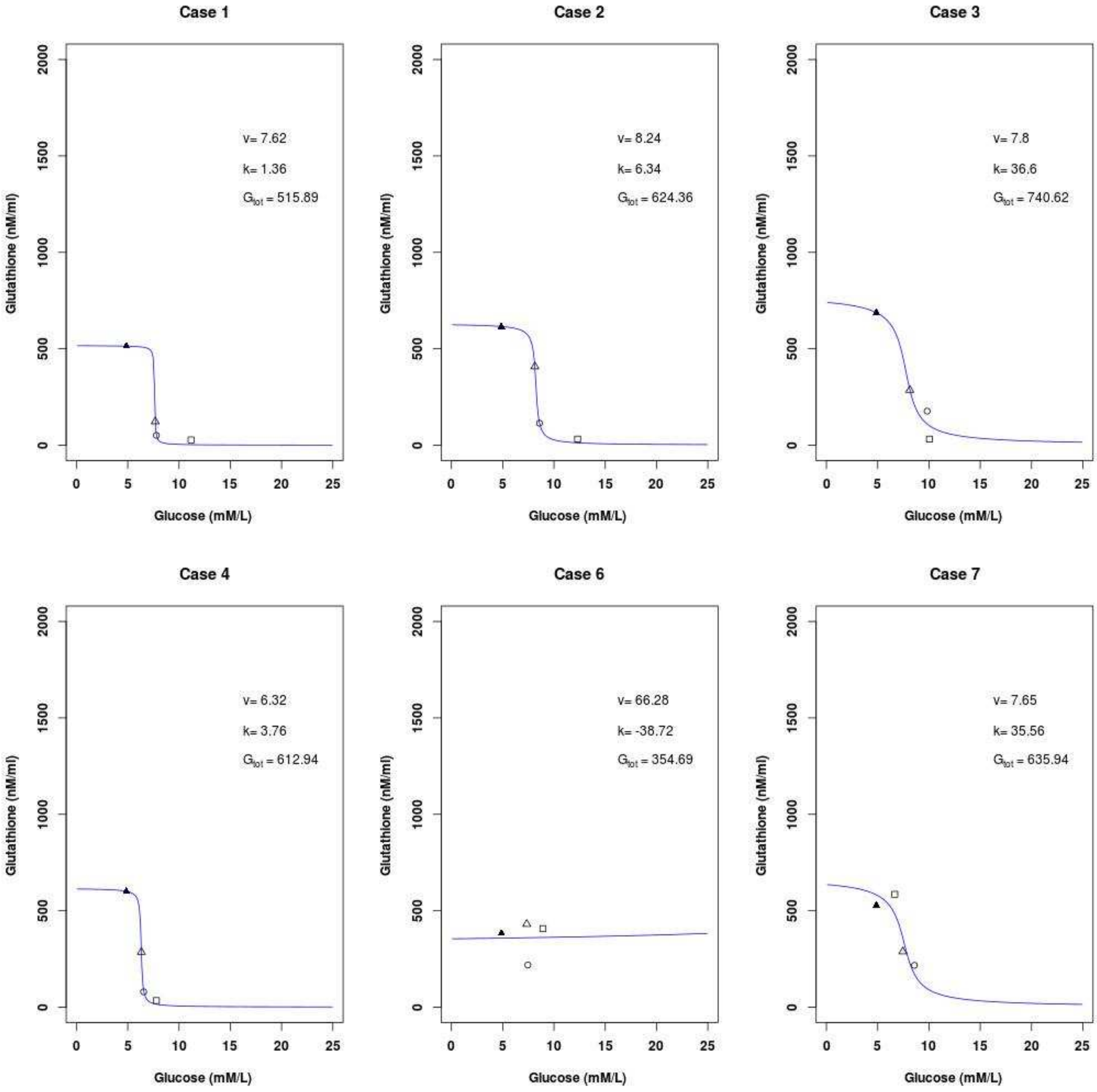

Figure S15: Individual sigmoid fits for diabetic cases 1-7. In each case the diabetic patient's glucose and GSH pair at 0 week (□), 4 weeks (○) and 8 weeks (△) are shown alongside glucose-GSH pair taken from non-diabetic subjects from their first visit (▲) using regression fit. The pathophysiological parameters v, k and G_{tot} estimated from a fit are displayed in its panel.

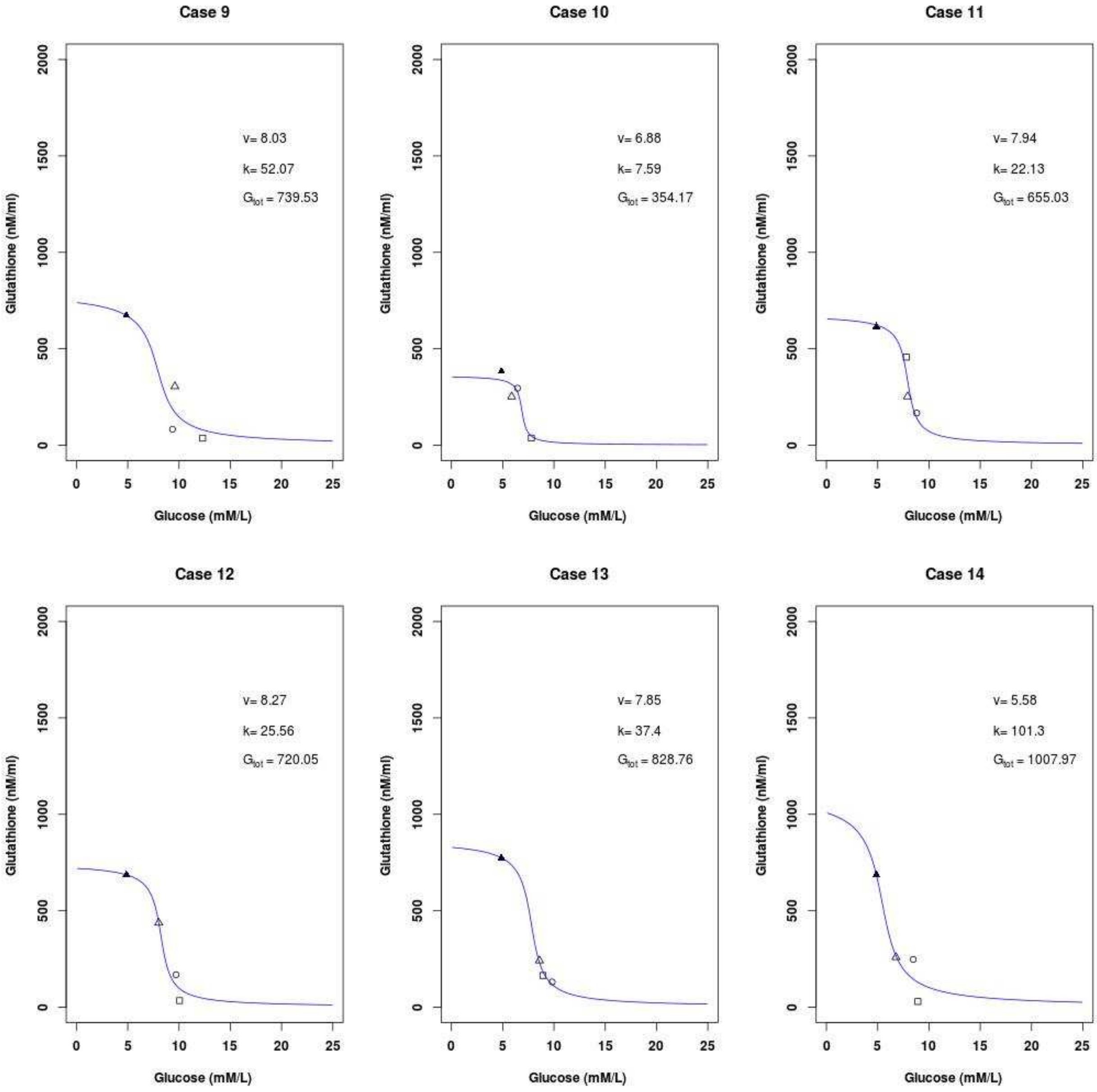

Figure S16: Individual sigmoid fits for diabetic cases 9-14. In each case the diabetic patient's glucose and GSH pair at 0 week (□), 4 weeks (○) and 8 weeks (△) are shown alongside glucose-GSH pair taken from non-diabetic subjects from their first visit (▲) using regression fit. The pathophysiological parameters v, k and G_{tot} estimated from a fit are displayed in its panel.

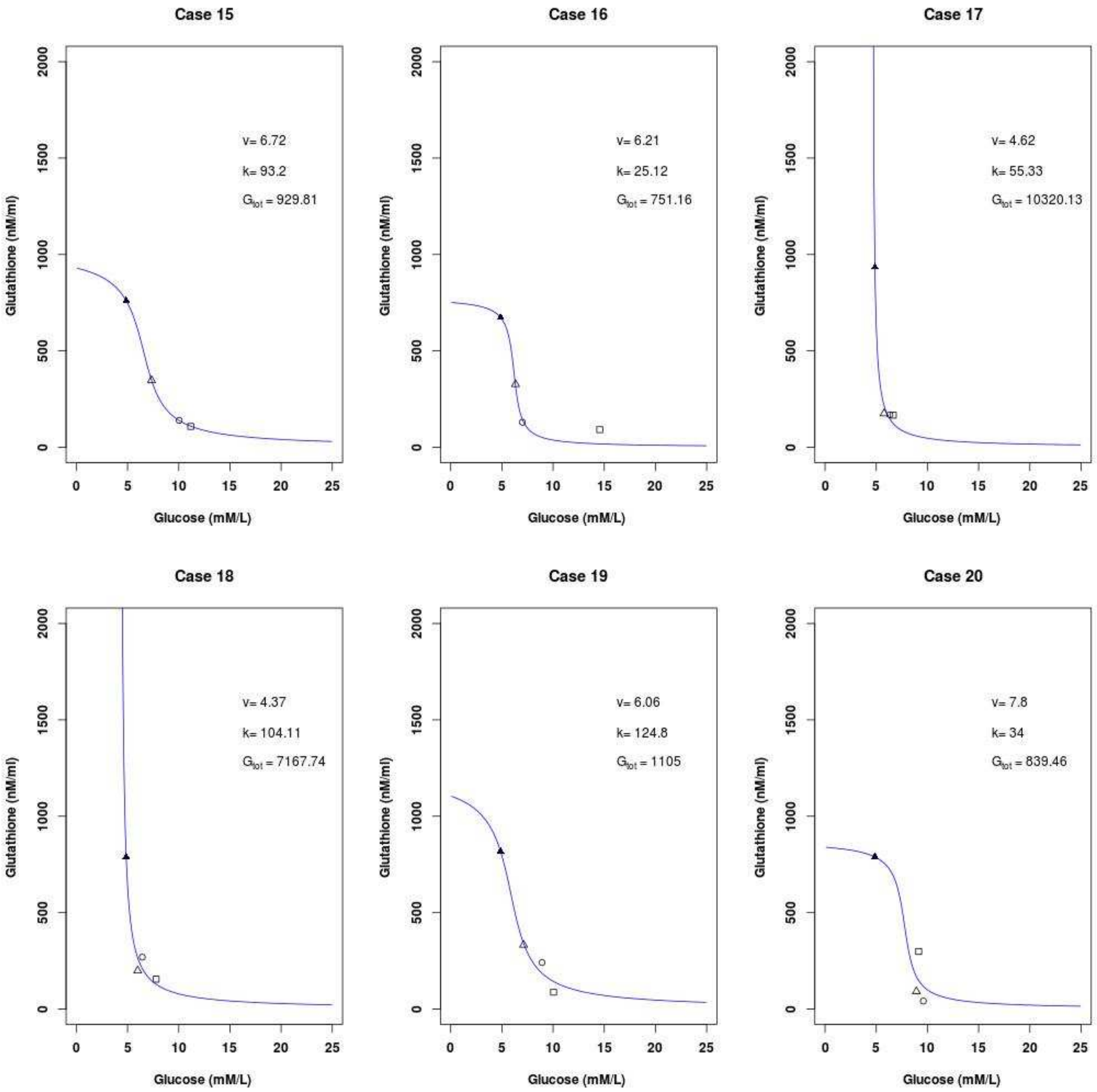

Figure S17: Individual sigmoid fits for diabetic cases 15-20. In each case the diabetic patient's glucose and GSH pair at 0 week (□), 4 weeks (○) and 8 weeks (△) are shown alongside glucose-GSH pair taken from non-diabetic subjects from their first visit (▲) using regression fit. The pathophysiological parameters v, k and G_{tot} estimated from a fit are displayed in its panel.

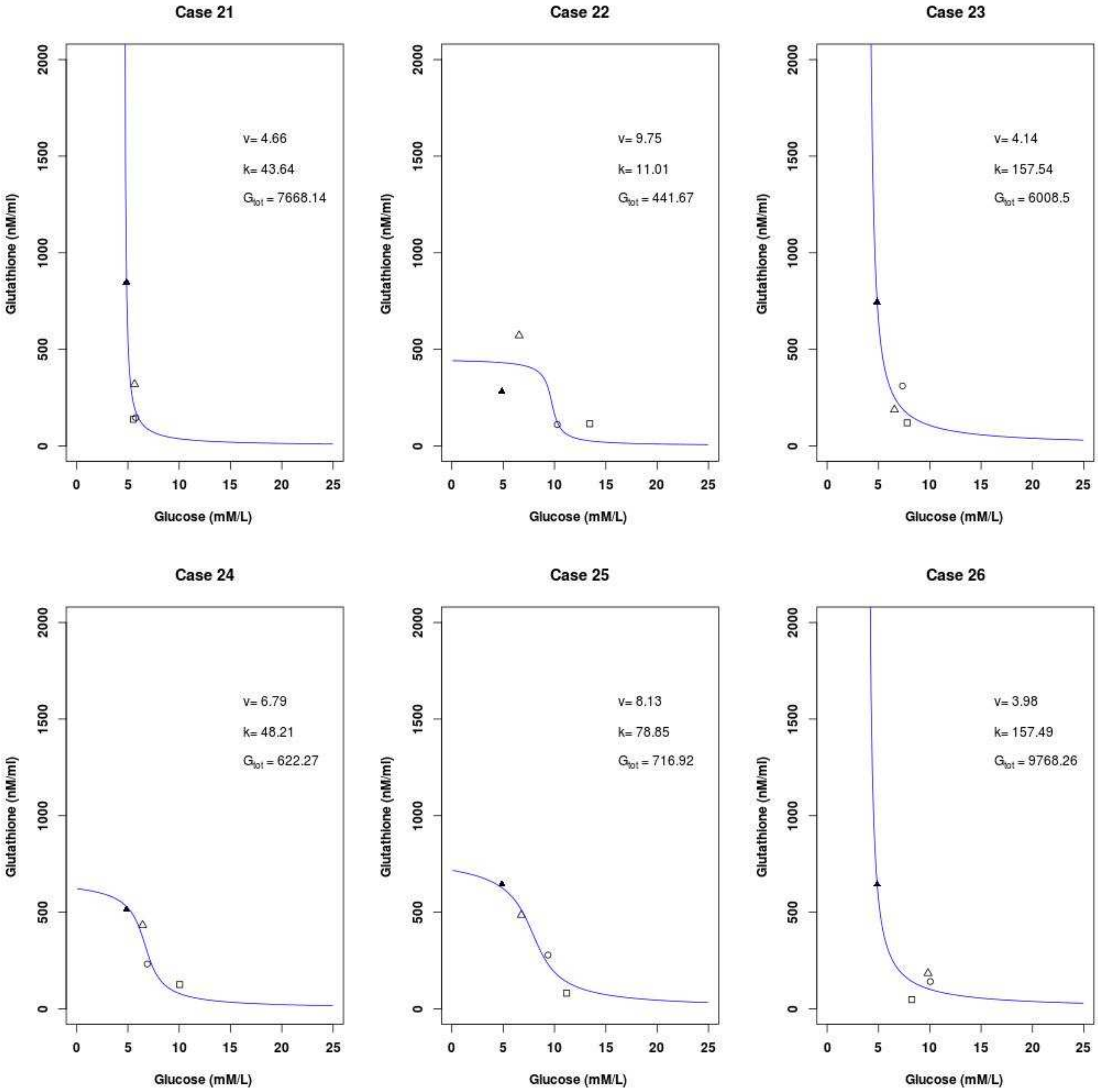

Figure S18: Individual sigmoid fits for diabetic cases 21-26. In each case the diabetic patient's glucose and GSH pair at 0 week (□), 4 weeks (○) and 8 weeks (△) are shown alongside glucose-GSH pair taken from non-diabetic subjects from their first visit (▲) using regression fit. The pathophysiological parameters v, k and G_{tot} estimated from a fit are displayed in its panel.

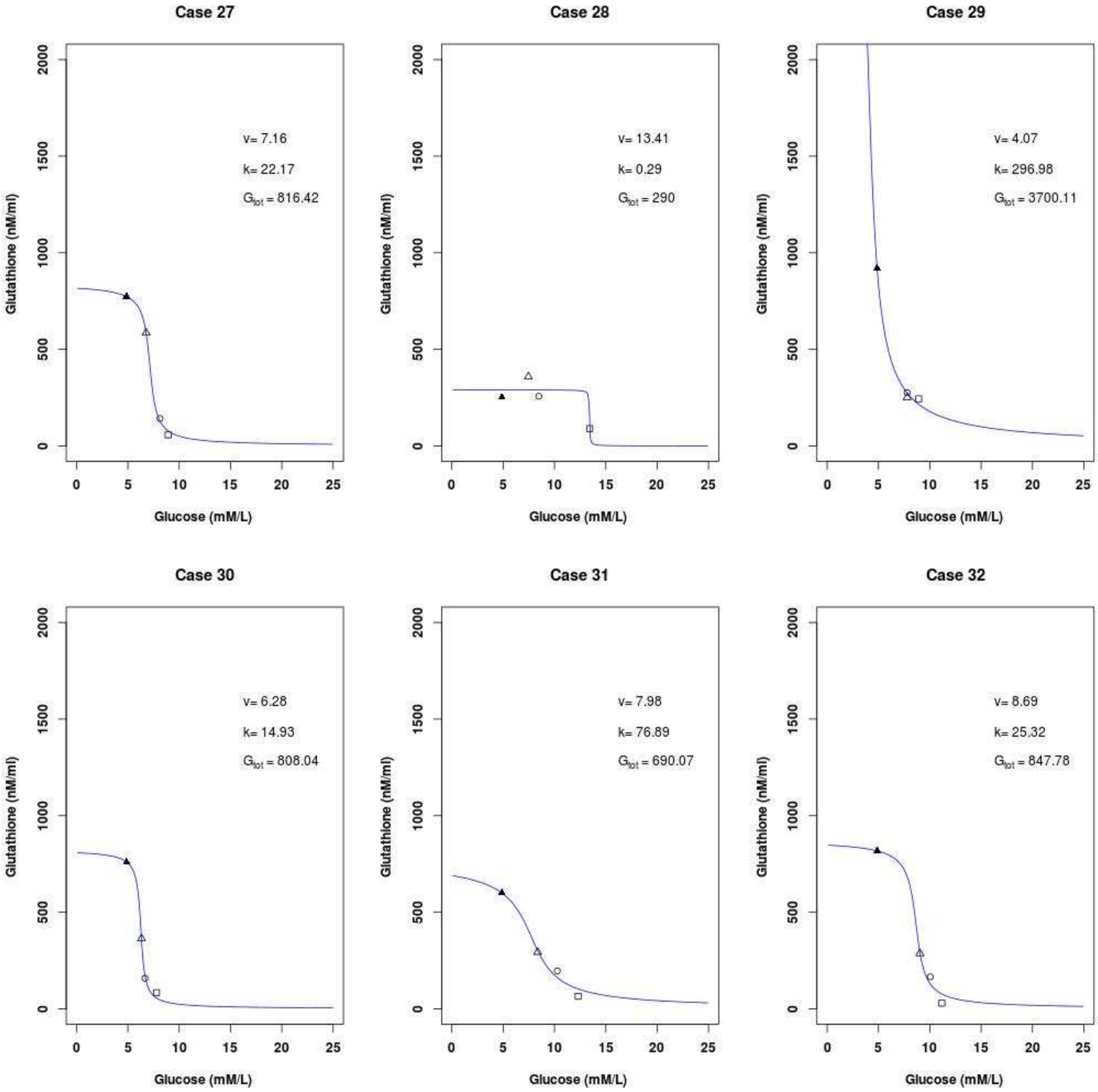

Figure S19: Individual sigmoid fits for diabetic cases 27-32. In each case the diabetic patient's glucose and GSH pair at 0 week (□), 4 weeks (○) and 8 weeks (△) are shown alongside glucose-GSH pair taken from non-diabetic subjects from their first visit (▲) using regression fit. The pathophysiological parameters v, k and G_{tot} estimated from a fit are displayed in its panel.

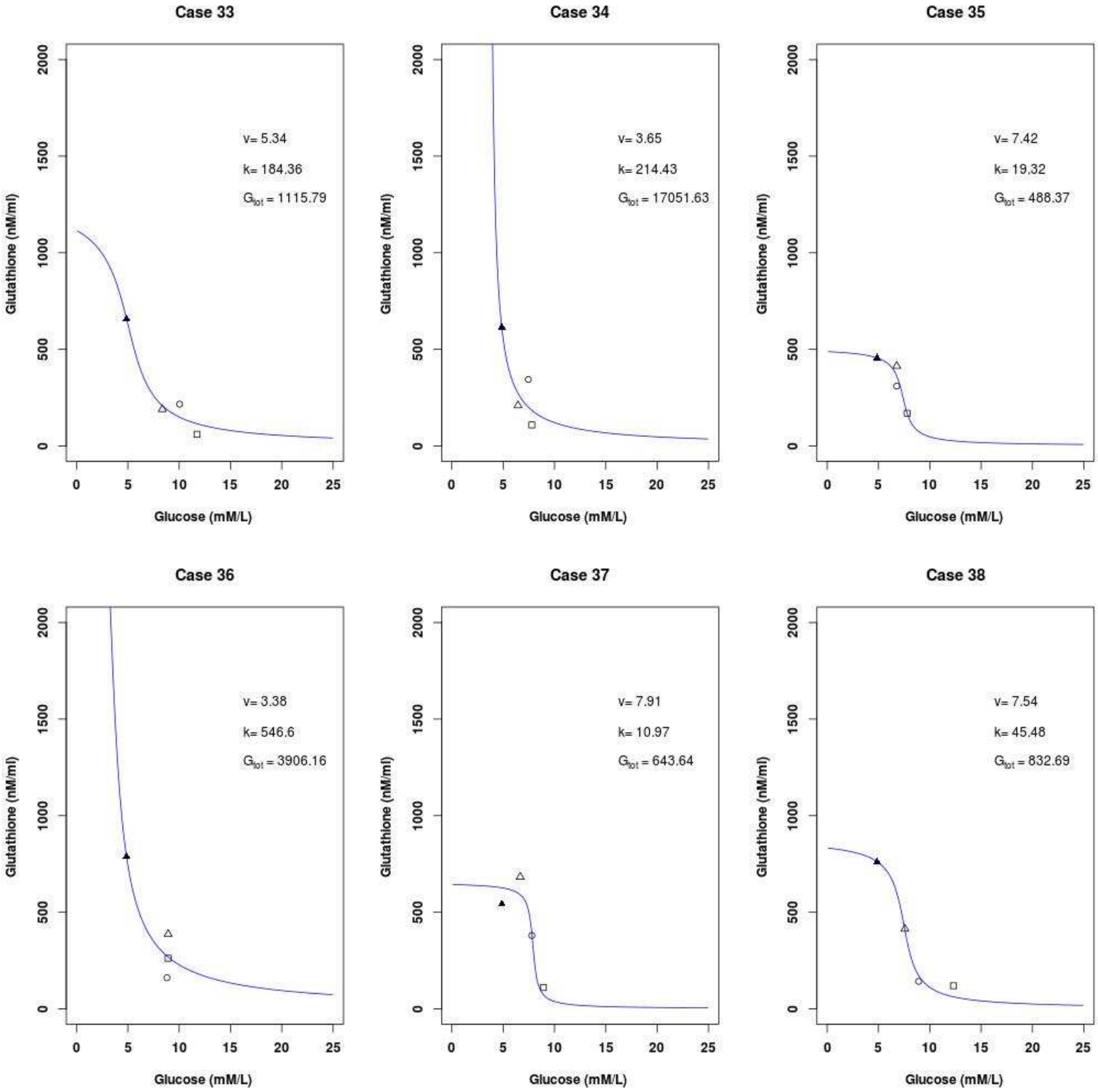

Figure S20: Individual sigmoid fits for diabetic cases 33-38. In each case the diabetic patient's glucose and GSH pair at 0 week (□), 4 weeks (○) and 8 weeks (△) are shown alongside glucose-GSH pair taken from non-diabetic subjects from their first visit (▲) using regression fit. The pathophysiological parameters v, k and G_{tot} estimated from a fit are displayed in its panel.

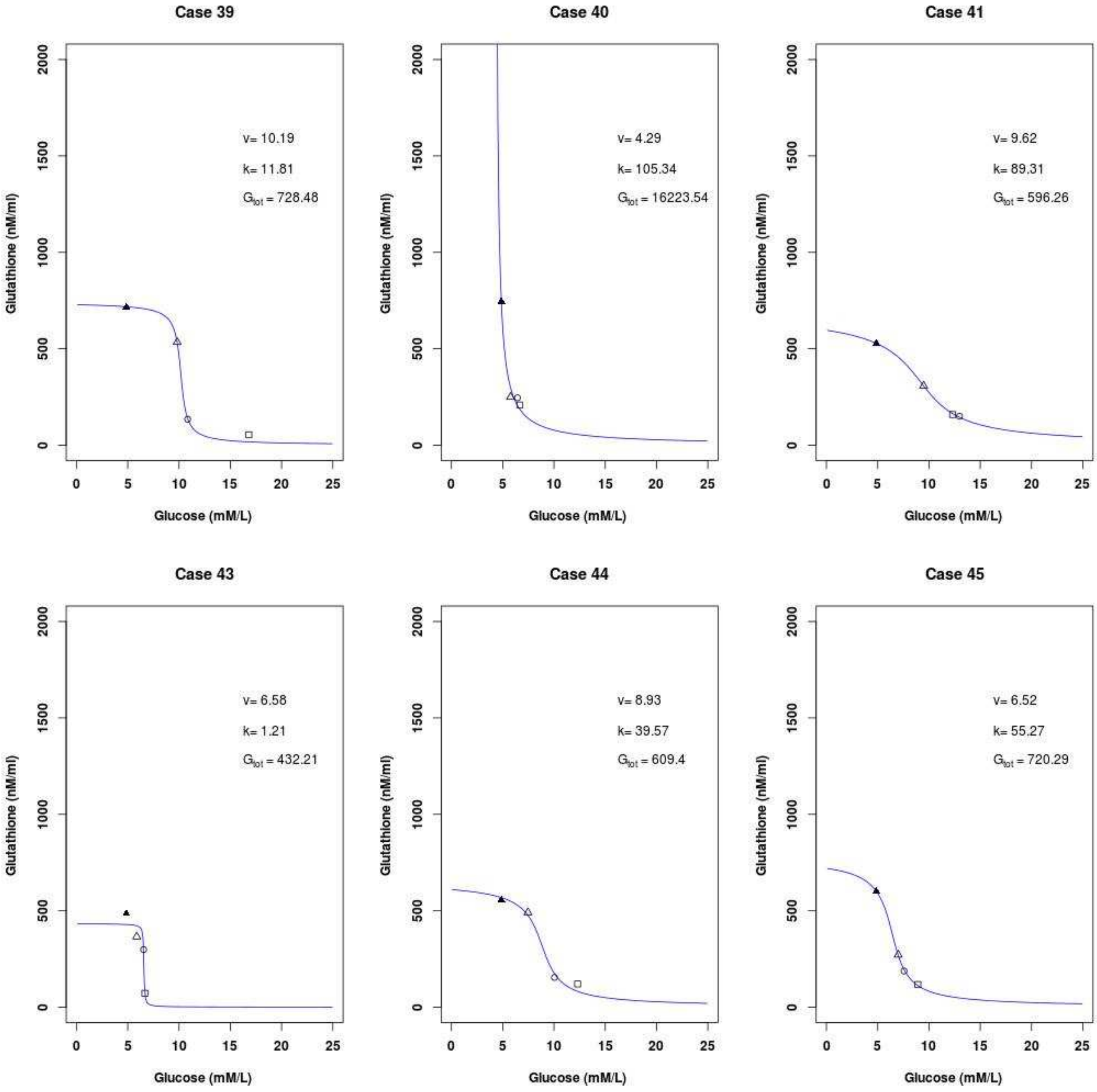

Figure S21: Individual sigmoid fits for diabetic cases 39-45. In each case the diabetic patient's glucose and GSH pair at 0 week (□), 4 weeks (○) and 8 weeks (△) are shown alongside glucose-GSH pair taken from non-diabetic subjects from their first visit (▲) using regression fit. The pathophysiological parameters v, k and G_{tot} estimated from a fit are displayed in its panel.

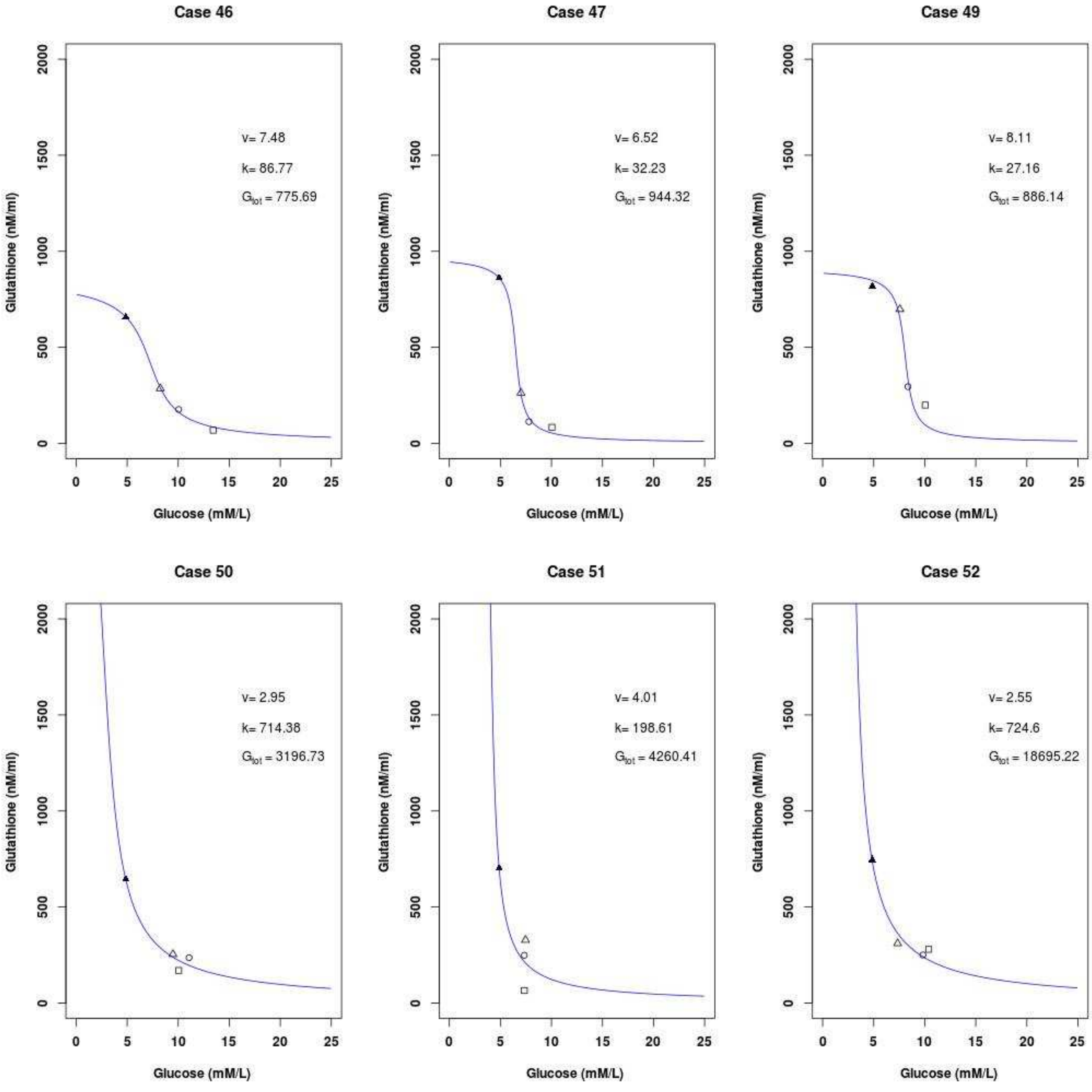

Figure S22: Individual sigmoid fits for diabetic cases 46-52. In each case the diabetic patient's glucose and GSH pair at 0 week (□), 4 weeks (○) and 8 weeks (△) are shown alongside glucose-GSH pair taken from non-diabetic subjects from their first visit (▲) using regression fit. The pathophysiological parameters v, k and G_{tot} estimated from a fit are displayed in its panel.

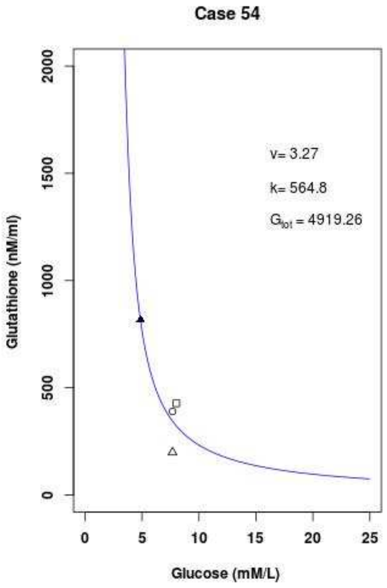

Figure S23: Individual sigmoid fits for diabetic case 54. In each case the diabetic patient's glucose and GSH pair at 0 week (□), 4 weeks (○) and 8 weeks (△) are shown alongside glucose-GSH pairs taken from non-diabetic subjects from their first visit (▲) using regression fit. The pathophysiological parameters v, k and G_{tot} estimated from a fit are displayed in its panel.

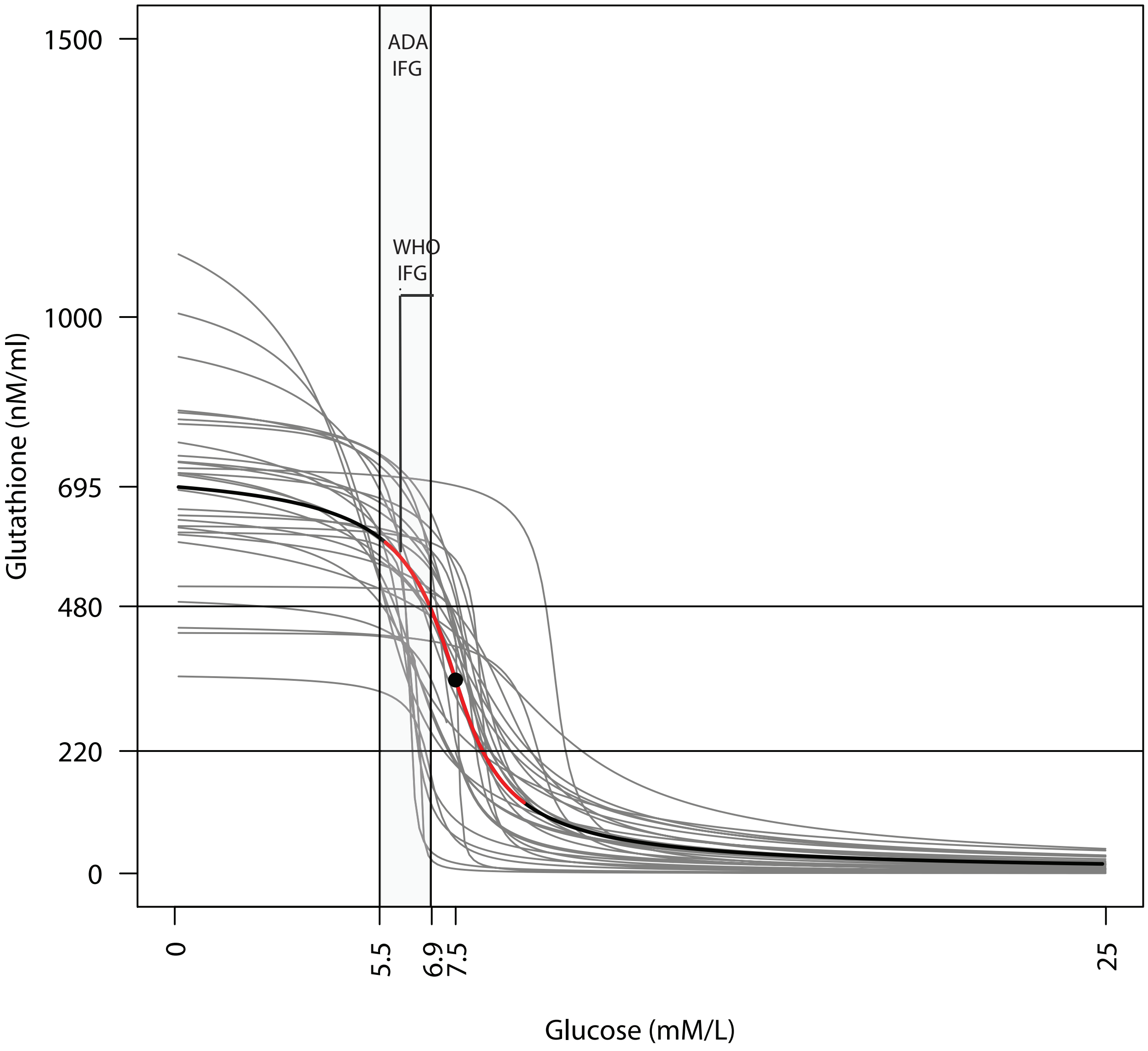

Figure S24: Individual response curves for diabetic patients above age 40 obtained using the minimal model are shown (thin gray lines, n= 29 out of 38) along with population-averaged curve (black bold line). The population-averaged curve has a threshold (black dot) at glucose = 7.5 mmol/L and GSH approximately 347; G_{tot}= 695 and k=43. An inflection regime is marked in red (width approximately one fourth of V, 1.87 mmol/L) is marked in red. The ADA impaired fasting glucose (IFG) range, 5.5-6.9 mmol/L and WHO IFG range 6-6.9 mmol/L is overlaid for the reference. The GSH band at 220-480 is the recovery phase for treated diabetics as shown in the main text, fig. 2. It is interesting to note that IFG occupies upper portion of the red curve, and 8-weeks patients lie in the lower portion of the red curve.

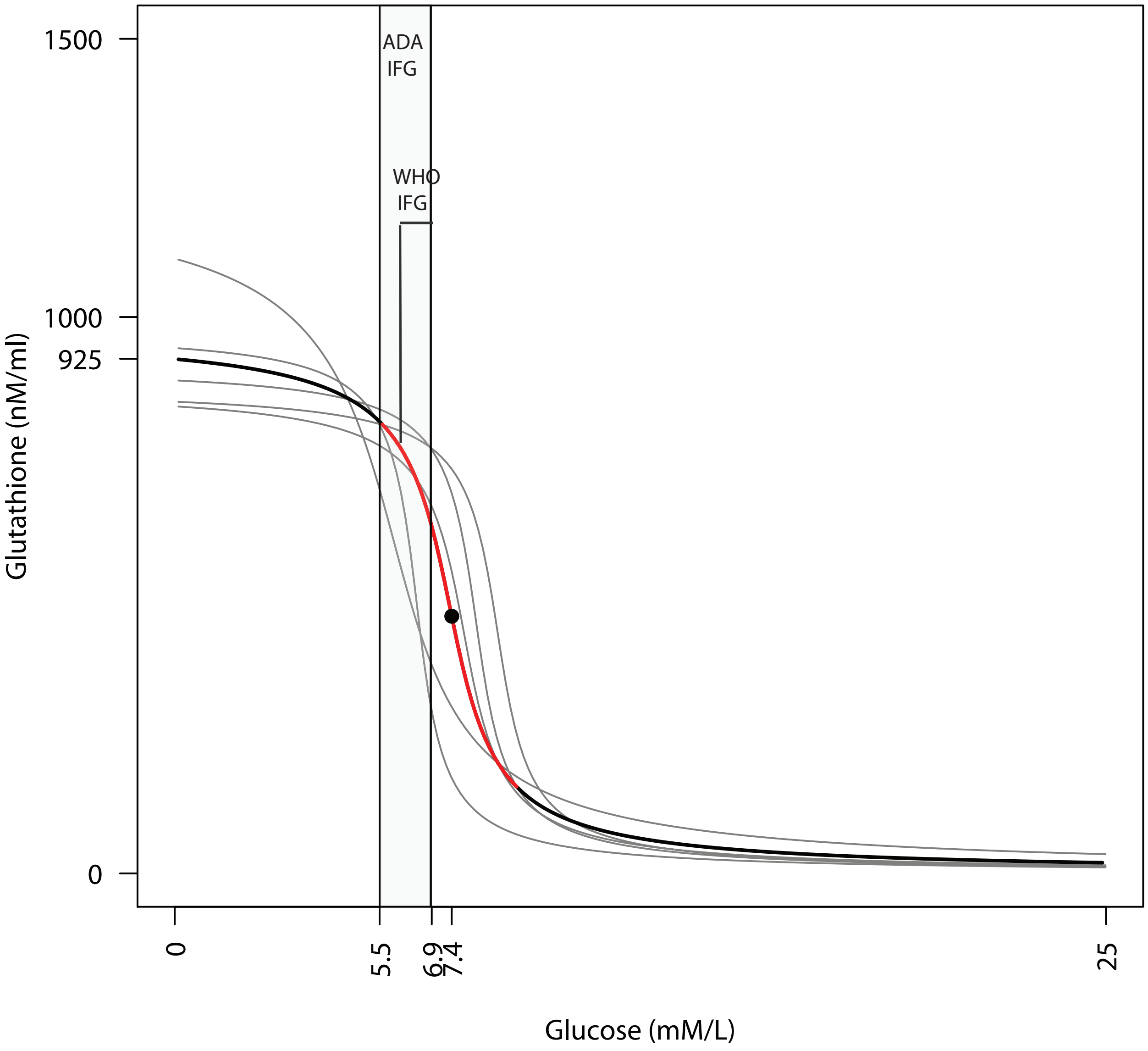

Figure S25: Individual response curves for diabetic patients below age 40 obtained using the minimal model are shown (thin gray lines, n= 5 out of 11) along with population-averaged curve (black bold line). The population-averaged curve has a threshold (black dot) at glucose = 7.4 mmol/L and GSH approximately 462; G_{tot}= 924 and k=48.7. An inflection regime is marked in red (width approximately one fourth of V, 1.85 mmol/L) is marked in red. The ADA impaired fasting glucose (IFG) range, 5.5-6.9 mmol/L and WHO IFG range 6-6.9 mmol/L is overlaid for the reference. Unlike the above 40 group, cluster analysis does not show GSH separation for diabetics recovery as shown in the figure S1. Nonetheless, the IFG band lies in the sensitive upper portion of the red curve.

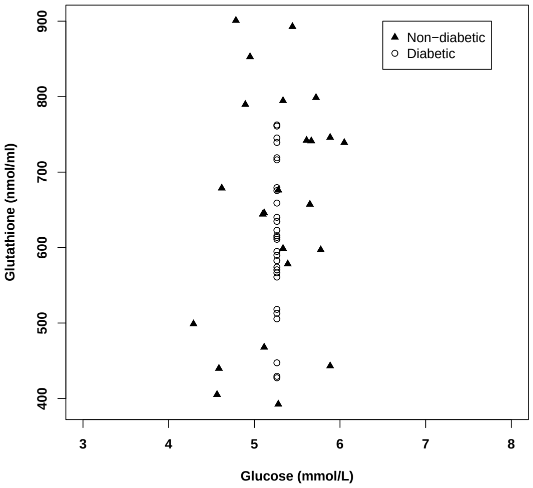

Figure S26: A comparison of GSH values of non-diabetic subjects and theoretical predictions of GSH values of diabetic patients at glucose were 5.2 mmol/L. This plot shows the natural variability in GSH at low glucose, in non-diabetics and diabetics.

different G_{tot} values, which explains this variation. To confirm this observation we computed the GSH values of diabetics at the average *non-diabetic* glucose value, 5.2 mmol/L. Figure S26 shows non-diabetic GSH values alongside the GSH value of each diabetic computed at 5.2 mmol/L from the fitted curve; the variations in the two groups are similar.

This implies that the variation in the GSH values in the non-diabetic population (as in the diabetic population, for that matter) arises from a natural variation in total GSH between individuals.

6 Distributions of the parameters v, k and G_{tot} in the diabetic population above age 40

Figure S27 shows the distributions of pathophysiological parameters v, k and G_{tot} of the 29 diabetics above age 40.

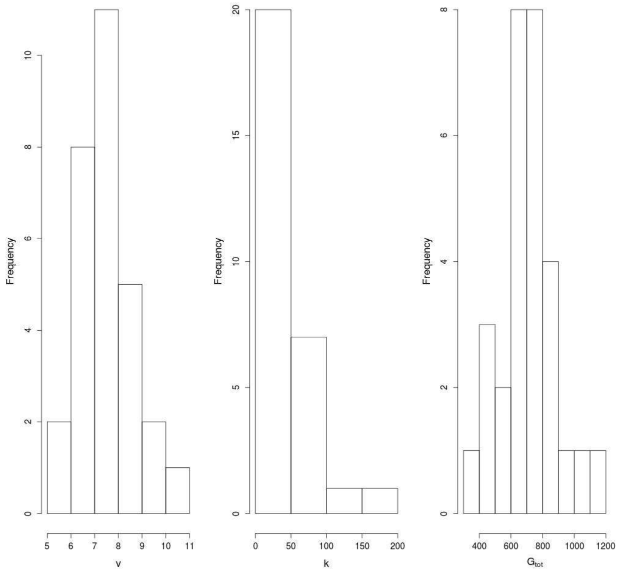

Figure S27: Distributions of v, k and G_{tot} in the diabetics above age 40. Mean and standard deviation values for v, k and G_{tot} are 7.5 ± 1.1, 43.0 ± 40.0 and 695 ± 166, respectively.

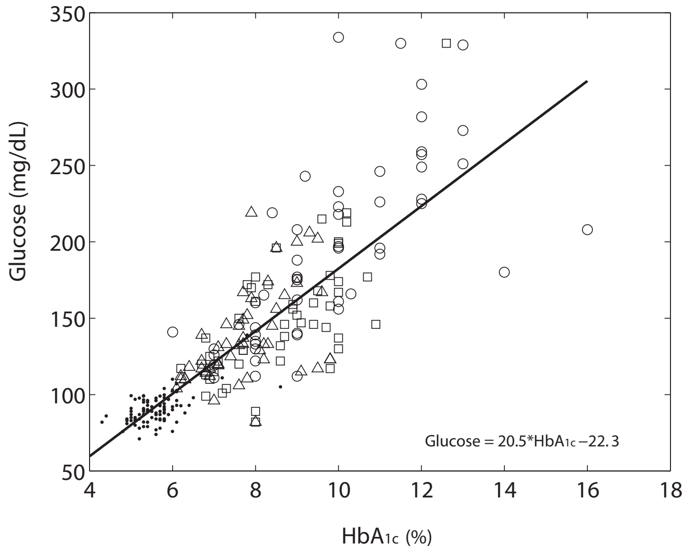

Figure S28: Linear regression between fasting glucose and HbA_{1c} . Fasting glucose and Hb1Ac values were taken from • : Non-diabetic; ∘: diabetic 0 week; □ : diabetic 4 weeks; △ : diabetic 8 weeks. This equation is used to convert HbA_{1c} into a glucose value for model fitting.

7 Estimation of the robustness of fits to the minimal model

In this section we study the robustness of the fitting procedures we have adopted. To obtain confidence that the individual fits are meaningful in the clinical context, we address two basic aspects of the fitting procedure:

1. How is variability in the glucose measurement of an individual accounted for in the model fitting?
2. How does daily variability in GSH of an individual affect the fits?

7.1 Accounting for variation in glucose levels in an individual

Glycated hemoglobin, HbA_{1c} , is a marker of average blood sugar level over approximately 3 months, and heavily weighted over the last 45-60 days. In contrast to the blood sugar levels glycated hemoglobin level is therefore stabler to fluctuations. In order to reduce variation in the fits we chose to model GSH against HbA_{1c} , not fasting glucose. Linear regression showed (Figure S28) that the relationship between fasting glucose and HbA_{1c} is

$$Glucose = 20.5\ HbA_{1c} - 22.3. \tag{15}$$

We converted HbA_{1c} levels of diabetics into equivalent glucose levels using Eq. (15) and used those in the fitting.

As is now common in the clinical setting, especially when modern facilities for HbA_{1c} values are available, our method uses HbA_{1c} for fits. The estimates are therefore reliable with respect to glucose measurements.

7.2 Accounting for the daily variation in GSH levels

GSH levels have been shown to vary 15-30% through the day [3]. Therefore, we ask: If we were to acknowledge that the measured GSH levels are uncertain to this extent, what would be the variability in v, k and G_{tot} obtained from fitting?

We generated an artificial 15% and 30% variation in the GSH data at 0, 4 and 8 weeks for diabetic Cases 13 and 15 (both age above 40), respectively, using a random number generator in R. 1000 such datasets were created in this manner for each Case, and each dataset was re-fitted. This generates distributions of

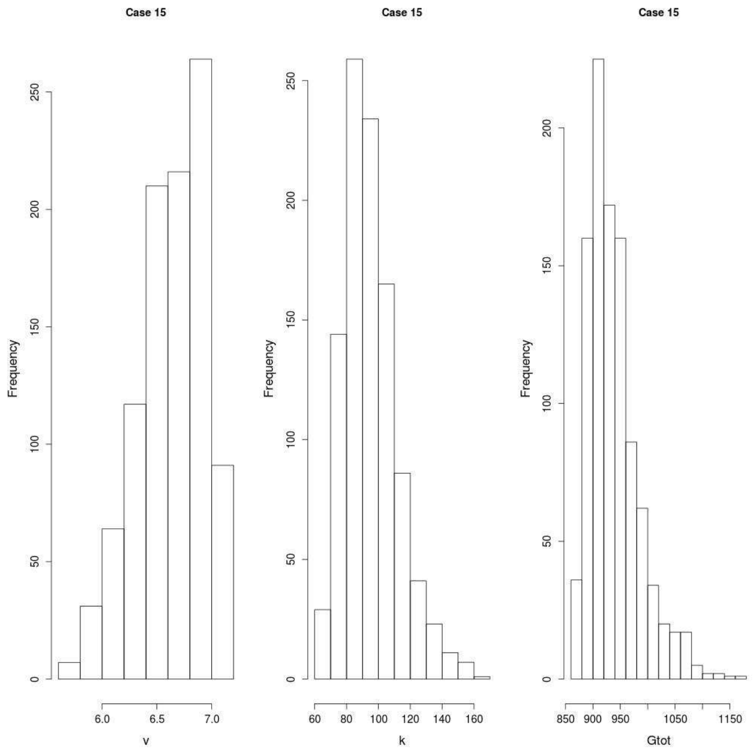

(a) Distributions of v, k and G_{tot} for Case 15 assuming an error in the measured GSH. GSH measurements at the 0, 4 and 8 week values were randomly varied up to 30%; 1000 datasets were generated and re-fit. The means and standard deviations for v, k and G_{tot} across these 1000 computations are 6.6 ± 0.3, 95.3 ± 16.7 and 938 ± 46, respectively.

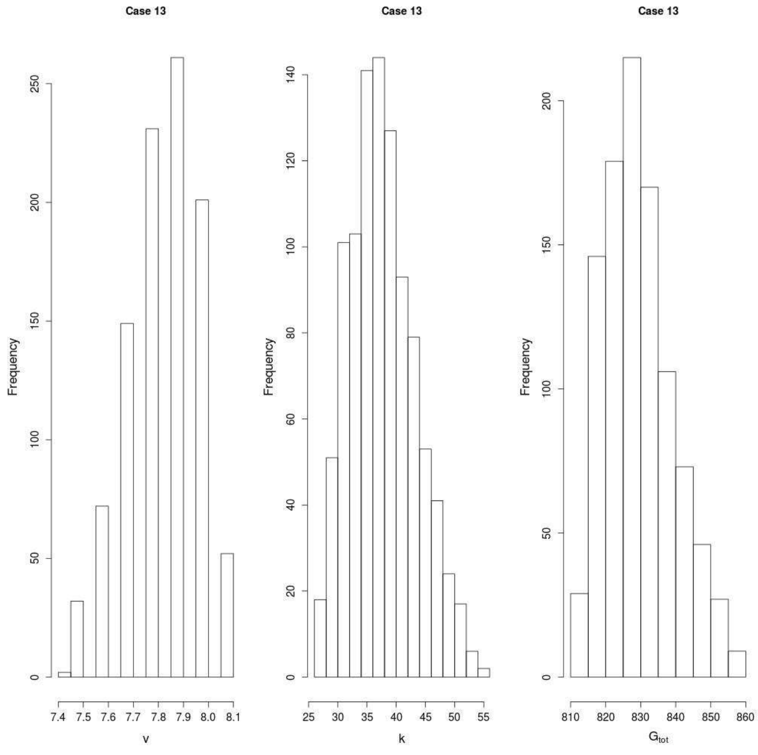

(b) Distributions of v, k and G_{tot} for Case 13 assuming an error in the measured GSH. GSH measurements at the 0, 4 and 8 week values were randomly varied up to 15%; 1000 datasets were generated and re-fit. The means and standard deviations for v, k and G_{tot} across these 1000 computations are 7.8±0.1, 37.7±5.6 and 829 ± 9, respectively.

Figure S29: Distributions of the parameters v, k and G_{tot} for the samples cases 15 and 13.

v, k and G_{tot} for the two sample Cases 15 and 13, Figures S29(a)and S29(b), respectively. The standard deviations in the parameters v, k and G_{tot} arising thus from a daily variation in GSH are much smaller than the standard deviations in the v, k and G_{tot} values across the population as shown in the figure S27. We conclude that the curve fitting is robust to diurnal GSH variation.

Taken together, these analyses suggest that any natural variability of glucose and GSH only somewhat affects the fits. Our method therefore provides reliable estimates of v, k and G_{tot}.

References

[1] Reed, M.C.,Thomas,R.L.,Pavisic,J.,James,S.J., Ulrich,C.M. and Nijhout,H.F. *A mathematical model of GSH metabolism*, Theor Biol Med, 5, 2008.

[2] Tyson,J.J.,Chen,K.C. and Novak,B.*Sniffers, buzzers, toggles and blinkers: dynamics of regulatory and signaling pathways in the cell*,Curr. Opin. Cell Biol., 15 (2), 2003.

[3] Blanco,R.A.,Ziegler,T.R.,Carlson,B.A.,Cheng,P.Y.,Park,Y.Cotsonis,G.A.,Accardi,C.J. and Jones,D.P.*Diurnal variation in GSH and cysteine redox states in human plasma*, Am. J. Clin. Nutr., 86 (4), 2007.

[4] Matthews,D.R.,Hosker,J.P.,Rudenski,A.S.,Naylor,B.A.,Treacher,D.F.and Turner,R.C. *H*omeostasis model assessment: insulin resistance and B-cell function from fasting plasma glucose and insulin concentrations in man Diabetologia; 28, 1985.

[5] Levy, J.C., Matthews,D.R.,Hermans,M.P. *C*orrect Homeostasis Model Assessment (HOMA) Evaluation uses the computer program Diabetes Care, 21, 1988.
